# Local and teleconnected temperature effects of afforestation and vegetation greening in China

**DOI:** 10.1093/nsr/nwz132

**Published:** 2019-09-12

**Authors:** Yue Li, Shilong Piao, Anping Chen, Philippe Ciais, Laurent Z X Li

**Affiliations:** 1 Sino-French Institute for Earth System Science, College of Urban and Environmental Sciences, Peking University, Beijing 100871, China; 2 Key Laboratory of Alpine Ecology and Biodiversity, Institute of Tibetan Plateau Research, Chinese Academy of Sciences, Beijing 100085, China; 3 Center for Excellence in Tibetan Earth Science, Chinese Academy of Sciences, Beijing 100085, China; 4 Department of Biology, Colorado State University, Fort Collins, CO 80523, USA; 5 Laboratoire des Sciences du Climat et de l’Environnement/Institut Pierre Simon Laplace, Commissariat à l’Énergie Atomique et aux Énergies Alternatives–CNRS–Université de Versailles Saint-Quentin, Université Paris-Saclay, F-91191 Gif-sur-Yvette, France; 6 Laboratoire de Météorologie Dynamique, Centre National de la Recherche Scientifique, Sorbonne Université, Ecole Normale Supérieure, Ecole Polytechnique, 75252 Paris, France

**Keywords:** afforestation, vegetation greening, China, biophysical climate effect, coupled land-atmosphere model

## Abstract

Afforestation in China provides carbon sequestration and prevents soil erosion, but its remote impacts on climate in other regions via the coupling of forest energy fluxes with atmospheric circulation are largely unknown. Here, we prescribe inventory-based forest cover change and satellite-observed leaf area index from 1982 to 2011 in a coupled land-atmosphere model to simulate their biophysical climate effects. Both local and global surface air temperatures show a seasonal contrast in response to past vegetation cover expansion over China: a phenomenon we primarily attribute to a variation of seasonality of vegetation greening. A large cooling in spring results in concurrent decreases in geopotential height over China and zonal wind over Mongolia, causing a dipole structure in the upper troposphere over the Arctic. This accounts for ∼58% of simulated spring warming over the Russian Arctic and ∼61% of simulated spring cooling over the Canadian Artic. Our results imply that spring vegetation dynamics in China may affect climate in northern high latitudes.

## INTRODUCTION

Large-scale afforestation programs were implemented in China during the past four decades and constitute the world’s most important efforts in restoring and expanding forests [1,2]. As a result, forest coverage in China increased to 21.6% by the early 2010s, a rise of 9.6% compared to the early 1980s [3]. This augmentation of forest coverage in China has strong impacts on local ecosystem services and climate, as it increases carbon sequestration [4,5], reduces soil erosion [6], prevents soil acidification in northern China [7] and cools local climate through increased surface evapotranspiration (ET) [8]. Large-scale afforestation also can have far-reaching ecosystem and climate effects beyond the geographical boundaries of the planted forests, primarily through its impacts on large-scale atmospheric circulations [9,10]. The scope of such teleconnected effects depends on the region considered and on the background climate [11–13]. As a result, observed local climate anomalies attributed to afforestation are a sum of both its immediate local effect and the induced large-scale climate feedback. For instance, global effects of afforestation (i.e. through atmospheric circulation, cloud cover and water vapor content) [13] often combine with local-scale surface energy balance changes (i.e. through alteration of the surface albedo, α, ET and roughness) [14,15] in controlling variations of surface air temperature (T_a_).

However, quantifying these local and teleconnected climate effects of afforestation on T_a_ can be a daunting challenge. Satellite remote sensing and eddy-covariance data provide an empirical approach, providing observational evidence to allow us to estimate the local biophysical effects of forest on climate at the surface [8,16–18]. For example, Peng *et al.* [8] revealed a local decrease of land surface temperature (LST) over afforestation areas compared to nonafforestation areas in southern China, which was attributed to higher surface ET caused by afforestation. The empirical approach used by Peng *et al*., however, may be insufficient to understand temporal changes of vegetation biophysical effects, because its space-for-time assumption neglects temporal (including seasonal, interannual and long-term) changes in forest structure (i.e. leaf area) and eventual shifts between energy-limited and water-limited ET regimes [19–21]. Furthermore, despite recently developed advanced diagnostic methods that attempt to isolate the year-to-year signal of local surface biophysical feedback of forests [19,22], it is still difficult for data-driven analyses to relate changes of LST into T_a_ [23] effects and to clearly identify the causal links between vegetation and climate [24,25]. In addition, previous studies rarely have considered the surface biophysical climate effects attenuated and extended beyond regions of vegetation change by large-scale feedback on atmospheric circulation [12]. Coupled land-surface climate models are useful numerical simulation tools with which to tackle the challenge of quantifying the long-term surface and large-scale atmospheric biophysical effects of vegetation cover. However, land surface models have been criticized for their inability to accurately simulate leaf area index (LAI) [26] and, hence, the LAI-dependent partition of surface net radiation (R_net_) into latent heat flux (LE) and sensible heat flux (Hs) [27]. These drawbacks partly can be overcome by prescribing satellite-observed LAI [28] in the models and optimizing the model parameters controlling the evaporative fraction against eddy-covariance observations [29].

We employ a global land-atmosphere–coupled climate model (the Institute Pierre Simon Laplace climate model, IPSLCM [30]) with simulations prescribed and constrained by empirical data from satellite and eddy-covariance observations to investigate how forest cover and LAI changes in China [31] have impacted the changes of T_a_ and surface energy balance in China and over remote regions over the last 30 years. The model grid is spatially variable, with a high spatial resolution zoom over China (central resolution of ∼1^o^ × 0.6^o^) and a coarser spatial resolution (∼4^o^ × 2^o^) for the outer areas. We perform two sets of experiments with the model: (i) a dynamic vegetation experiment (SCE) in which the model is forced with observed LAI, forest cover fraction constructed from inventory data, historical sea surface temperature (SST) and carbon dioxide (CO_2_) concentration from the period 1982–2011 and (ii) a control experiment (CTL) where the SST and CO_2_ forcing data are identical to SCE, but LAI data are fixed by the seasonal climatology and forest cover remains static at its 1982 value for each grid cell in China throughout the experimental period of 1982–2011 (Methods). This experiment design builds on Li *et al*. [32] by extending the model simulations of a 15-member ensemble into 30 members (different members have different atmospheric initial conditions). Here, we first evaluate the performance of model-simulated T_a_ and analyze local vegetation biophysical feedback, including their spatial and seasonal variations. We then explore the teleconnections between vegetation cover change in China and the climate in remote regions. Bringing observed LAI and forest cover into a climate model enables us partly to overcome the limitations of local-scale studies based on, for example, satellite and flux-tower data [8] that document local climate effects and consider the large-scale climate effects resulting from LAI and forest increases in China.

## RESULTS

### Evaluations of simulated temporal variations in T_a_ and surface energy flux over China

**Figure 1.**
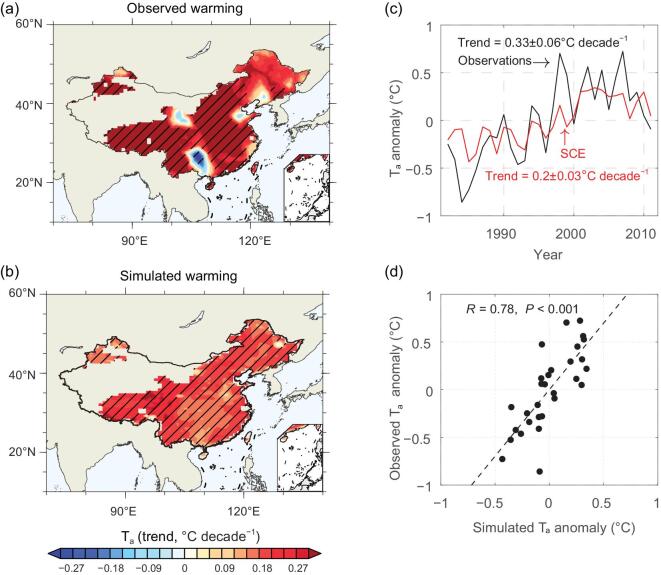
Observed and simulated warming in China from 1982 to 2011. The trend in annual mean air temperature (T_a_) over China was computed from (a) an observed data set from the Climate Research Unit (CRU TS3.21) [33] and (b) model simulations from the experiment SCE corresponding to their (c) time series of T_a_ anomaly (minus 30-year mean) and (d) a scatter plot of observed versus simulated T_a_ anomaly. The area with climatological leaf area index less than 0.1 was masked, and hatching indicates a 95% confidence level.

Figure 1 shows the comparison between observed and simulated temporal variations of annual mean T_a_ in China from 1982 to 2011. Observed temperature from the Climate Research Unit (CRU) [33] shows significant warming with a mean value of 0.33 ± 0.06°C decade^−1^ (*P* < 0.001) across almost the whole country, except for Northeast China and the central Yunnan-Guizhou plateau (Fig. 1a). The overall warming pattern, as well as the variability of T_a_, is reproduced generally well by the SCE simulations, albeit with an underestimation of the warming rate (Fig. 1b). The underestimated interannual variance is partly a consequence of the multi-member ensemble average (Fig. 1c and d). In addition, the inability of the model to reproduce the interannual amplitude of winter T_a_ also may contribute to the underestimation of the interannual variance of T_a_ (Supplementary Fig. 1b, f). In general, the IPSLCM model reasonably reproduces the observed climate warming during the vegetation growing season (e.g. from April to October) (Supplementary Fig. 1a, c, d).

The model’s performance in simulating surface radiation fluxes was evaluated against satellite observations from the Clouds and the Earth’s Radiant Energy System (CERES) SYN1deg-Month [34] available for 2001–2011, reanalysis products from the European Centre for Medium-Range Weather Forecasts (ECMWF, ERA-Interim) [35] and the National Centers for Environmental Prediction (NCEP) [36] available for the period 1982–2011 (Supplementary Figs 2 and 3). The model captures the spatial patterns and seasonal variations of downward shortwave (S_in_) and longwave radiation (L_in_), but there is a positive bias of S_in_ in southern China across all seasons that may lead to overestimation of solar energy absorbed by forests (Supplementary Fig. 2). For temporal variations, the model fails to simulate the decadal trend of observed S_in_ and L_in_ (Supplementary Fig. 3a, b), possibly because it lacks the radiative forcing of aerosols and other short-lived forcing. Nevertheless, the interannual variability of S_in_ is captured and, in particular, compares well to data from ERA-Interim and NCEP (Supplementary Fig. 3a). The observed temporal variation of surface turbulent fluxes, such as the increase in LE over China through enhanced vegetation activity, also is simulated reasonably well by the model, as shown in Li *et al*. [32] (see also Supplementary Fig. 3e and Table 1).

### Simulated biophysical effects due to afforestation and vegetation greening in China

**Figure 2.**
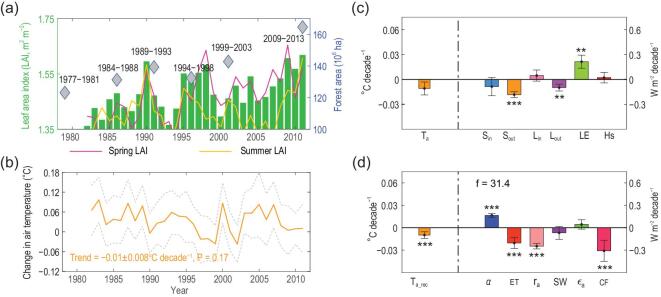
Temporal change in vegetation cover and impact on surface air temperature and energy balance in China from 1982 to 2011. (a) Time series of the April-to-October mean satellite-based leaf area index (LAI, green bars) and inventory-based forest area across the country. Temporal variations in spring (March, April and May, magenta line) and summer (June, July and August, yellow line) LAI also are shown and offset by 0.8 m^2^ m^−2^ and 1.8 m^2^ m^−2^, respectively. (b) Temporal change in surface air temperature (T_a_) is shown by the orange curve represented by SCE minus CTL (hereafter, vegetation-induced, see Methods). Uncertainty is quantified by computing the standard deviation from 100 combinations with each combination containing 10 members that are selected randomly from the 30 members. (c) Vegetation-induced trend in simulated T_a_ and energy fluxes, including downward (S_in_) and upward (S_out_) shortwave radiation, downward (L_in_) and upward (L_out_) longwave radiation, latent (LE) and sensible (Hs) heat fluxes. (d) Vegetation-induced trend in the reconstructed T_a_ (T_a_rec_) and vegetation-induced changes in surface energy balance related to change in surface albedo (α), evapotranspiration (ET), aerodynamic resistance (r_a_), shortwave radiation (SW), air emissivity (ε_a_) and the combined forcing and response (CF) (see Equation (28) in Methods). ***Significance at the 99% confidence level, **significance at the 95% confidence level and *significance at the 90% confidence level.

Figure 2a shows the vegetation cover increases across China due to growing-season greening [31] and the expansion of forest area [3] during the period 1982–2011. The signal due to vegetation biophysical effects can be isolated by subtracting the CTL variables from those of SCE (SCE - CTL; hereafter, vegetation-induced changes). We find that there is a significant negative correlation (*R* = −0.53, *P* < 0.01) between LAI and vegetation-induced change in T_a_, with higher LAI being associated with a decreased T_a_ (e.g. cooling effects), both during specific years (e.g. 1990 versus 1993) and for the overall trend (Fig. 2b). As a result, the simulations show large-scale afforestation and vegetation greening leading to a small reduction of T_a_ across China of −0.01 ± 0.008°C decade^−1^ (*P* = 0.17).

To further examine the mechanisms affecting T_a_, we computed the trend of vegetation-induced surface energy fluxes and decomposed the effect on T_a_ into separate factors following Zeng *et al*. [28] (Methods). The decomposition can reproduce the sign and magnitude of the vegetation-induced trend in T_a_ and its components due to different biophysical effects (Fig. 2c and d). Decreased α, induced by increased vegetation cover, has a positive forcing on T_a_ that significantly reduces surface upward shortwave radiation (S_out_) by −0.18 ± 0.07 W m^−2^ decade^−1^ (*P* < 0.001). In contrast, the vegetation-induced changes in S_in_ (−0.09 ± 0.02 W m^−2^ decade^−1^, *P* = 0.44) and L_in_ (0.05 ± 0.04 W m^−2^ decade^−1^, *P* = 0.48) are smaller and insignificant, indicating that changes in LAI and forest cover over China have negligible effects on these components. The decrease in upward longwave radiation (L_out_) is a response to surface cooling and has a trend of −0.10 ± 0.09 W m^−2^ decade^−1^ (*P* < 0.05).

The increase in the net radiation available for plants, R_net_, is due mainly to lower α, and the majority of this increase is used to enhance LE (0.21 ± 0.06 W m^−2^ decade^−1^, *P* < 0.05) (Fig. 2c). Because higher LAI increases roughness, the aerodynamic resistance (r_a_) also is reduced by −0.32 ± 0.05 s m^−1^ decade^−1^ (*P* < 0.05). Such a decrease of r_a_ is expected to increase the sensible heat flux, Hs (see Equation (4) in Methods), but the extent of its influence on ET is much less, because, in this case, the effects of the canopy [37] and stomatal resistance [38] of the vegetation dominate over r_a_. Although the climate response due to decreased r_a_ is larger than that due to enhanced ET (Fig. 2d), the increasing Hs (due to reduced r_a_) is offset by a simultaneous decline in the difference between surface temperature (T_s_) and T_a_. Another possible explanation of this result is that the term T_s_ − T_a_ was artificially split from the formulation of Hs to the left side of Equation (1) during the differentiation processes and the coefficient $\frac{\rho {C}_p}{r_a}$ is added as a part of the energy redistribution factor (*f*) (see Methods). The effects of reduced r_a_ on Hs are thus dampened by dividing the term related to the response of altered r_a_ by the increased *f* value. This effect is apparent in the model result of the rather flat trend of Hs induced by vegetation change over the last 30 years (Fig. 2c).

**Figure 3.**
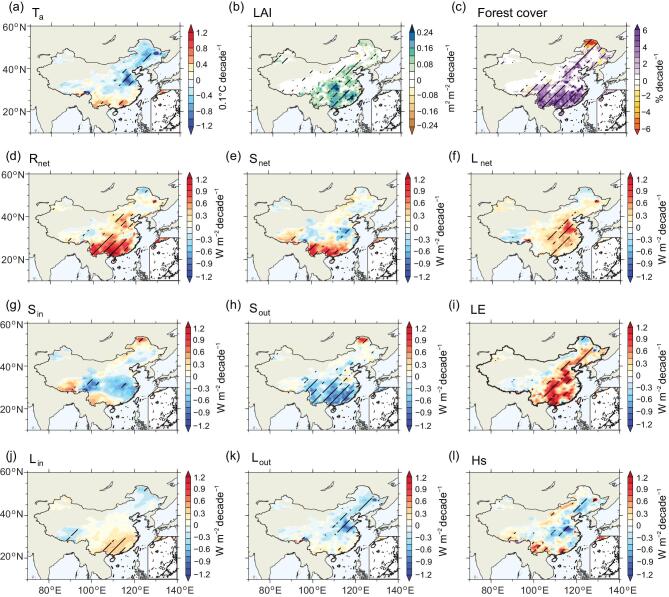
Spatial patterns of the trend in annual mean surface air temperature and energy fluxes induced by vegetation cover expansion in China from 1982 to 2011. (a, d–l) Surface air temperature (T_a_), net radiation (R_net_), net shortwave radiation (S_net_), net longwave radiation (L_net_), downward shortwave radiation (S_in_), upward shortwave radiation (S_out_), latent heat flux (LE), downward longwave radiation (L_in_), upward longwave radiation (L_out_) and sensible heat flux (Hs), respectively. (b, c) Trend in satellite-observed growing season (April-to-October mean) leaf area index (LAI) and inventory-based forest cover fraction. The linear trend is computed for SCE minus CTL (i.e. vegetation-induced, see Methods). The area with climatological leaf area index less than 0.1 was masked, and hatching indicates a 95% confidence level.

### Spatial variations of vegetation-induced change in T_a_ and surface energy balance

At the annual scale, the response of T_a_ to changes in vegetation structure and cover shows a pattern of northern cooling and southern warming in China (Fig. 3a). Cooling effects occur in North China and in the Northeast Plain, where forest cover fraction increased at a rate of 2% decade^−1^ and LAI at a rate of 0.08–0.12 m^2^ m^−2^ decade^−1^ (Fig. 3b, c). Yet, the largest increases in LAI (0.24 m^2^ m^−2^ decade^−1^) or in forest cover fraction (over 6% decade^−1^), which were observed in southern China (Fig. 3b, c), had only a small impact on T_a_. In the southwestern Yunnan province and the Pearl River deltas, vegetation changes are modelled to cause a warming effect (Fig. 3a), attributable to the increased R_net_ (contributions from both S_net_ and L_net_) and the lack of energy dissipation as suggested by the stable LE and increased Hs (Fig. 3d, e, f, i, l). In southern China, the modelling results indicated that the potential cooling effects of increased LE were offset by decreased S_out_ due to a lower α (Fig. 3h, i).

Compared to surface energy balance changes, large-scale atmospheric biophysical feedback seem to have a smaller effect on T_a_ than on surface energy flux changes. However, the two processes interact with one another. For example, there is a negative tendency of S_in_ caused by increased total cloud cover (not significant) that partly counteracts the decrease in S_out_ due to surface atmospheric cooling (e.g. in the Yangtze River basin [Fig. 3e, g, h]). Atmospheric circulation feedback also result in increased L_in_ that contributes to over 80% of the simulated increase in L_net_ in southeastern China (Fig. 3f, j). This spatial pattern of increased L_in_ is consistent with a vegetation-induced increase in column-integrated precipitable water (not shown), indicating a vegetation-induced increase in surface and upper-level water vapor in southeastern China.

Further decomposition (Equation (8) in Methods) confirms that the spatial pattern of vegetation-induced T_a_ change is primarily attributed to surface biophysical effects through decreased α and enhanced ET (Supplementary Fig. 4a, d, e, i). As described above, the effects of reduced r_a_ on Hs (Supplementary Fig. 4f) were dampened when divided by the increased *f* value. On the other hand, the large-scale biophysical effects through atmospheric changes on S_in_ and L_in_ may locally amplify or dampen the sum of these vegetation-induced changes in forcing and response (Supplementary Fig. 4c, g, h), which eventually is redistributed by the spatial pattern of *f* (Supplementary Fig. 4b).

### Seasonal dependency of local vegetation biophysical effects

**Figure 4.**
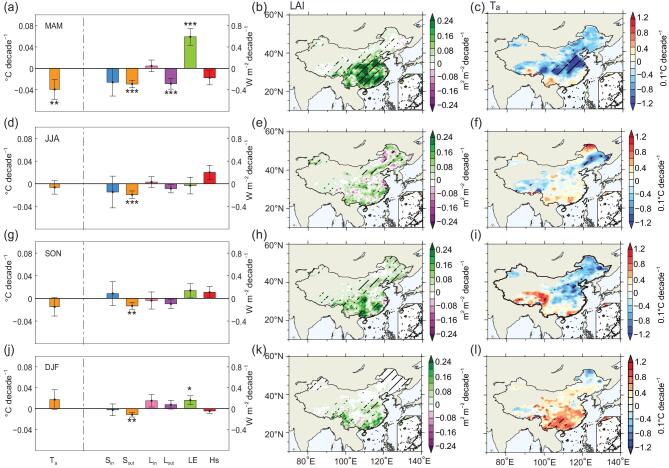
Seasonal dependency of the vegetation impacts on surface air temperature and energy fluxes in China from 1982 to 2011. (a, d, g, j) Vegetation-induced trends in simulated T_a_ and energy fluxes, which are the same as Fig. 2c but for spring (March, April and May, MAM), summer (June, July and August, JJA), autumn (September, October and November, SON) and winter (December, January and February, DJF), respectively. ^***^Significance at the 99% confidence level, **significance at the 95% confidence level and *significance at the 90% confidence level. (b, e, h, k) and (c, f, i, l) show the spatial patterns of the trend in satellite-observed LAI from Global Inventory Modeling and Mapping Studies (GIMMS) [76] and the vegetation-induced trend in surface air temperature (T_a_) during corresponding seasons, respectively. The area with climatological leaf area index less than 0.1 was masked, and hatching indicates a 95% confidence level.

In this section, we analyze the vegetation-induced biophysical effects over different seasons across China (Fig. 4). The largest vegetation impact on T_a_ is found in spring (i.e. from March to May), with the vegetation-induced T_a_ trend being a cooling of −0.04°C decade^−1^ (*P* < 0.05) (Fig. 4a). If this magnitude of cooling is compared to the simulated regional warming of about 0.16 ± 0.04°C decade^−1^ (*P* < 0.001) (Supplementary Fig. 1c), it is apparent that afforestation and vegetation greening has slowed down spring warming by about 25% in China during the past 30 years. This cooling trend mainly occurs in North China and the Yangtze River basin (Fig. 4c), indicating that enhanced spring LAI leads to increased LE under both water-limited (e.g. North China) and energy-limited (e.g. the Yangtze River basin) ET regimes [39] (Fig. 4a, b; Supplementary Fig. 5c). It also is found that decreasing S_in_, driven by increased cloud cover, counteracts the decrease of S_out_ in the Yangtze River basin (Supplementary Fig. 5a, b, i). To the south of the Yangtze River basin, the afforestation-induced decrease in α primarily explains the decrease in S_out_ and counteracts the cooling effect of LE enhancement (Fig. 4d; Supplementary Fig. 5b, c). Increased LE in spring results in a higher surface-specific humidity (Q2m) over both South and North China (Supplementary Fig. 5g). Further, vegetation-induced circulation changes strengthen southwesterly winds at 850 hPa, which is favorable for an increase in atmospheric water vapor over South China and the adjacent seas (Supplementary Fig. 5h). As a consequence of greater column-integrated water vapor content (i.e. precipitable water, by ∼0.2 mm decade^−1^) in southern China, surface L_in_ increases in this region due to the increased greenhouse effect (Supplementary Fig. 5d).

During the other three seasons, changes in T_a_ and energy fluxes are smaller than those in spring (Fig. 4d–l). In summer (i.e. from June to August), the only areas with significant greening are the Northeast China Plain, where a cooling effect is caused by enhanced LE (Fig. 4e, f; Supplementary Fig. 6c). Biophysical feedback from atmospheric circulation only emerge over Southeast China, where total cloud cover increases (Supplementary Fig. 6i) and S_in_ decreases, counteracting the decreased S_out_ (Fig. 4d; Supplementary Fig. 6a, b). In autumn (i.e. from September to November) and winter (i.e. from December to February), vegetation-mediated changes in T_a_ are not significant across the whole country (Fig. 4i, l), with opposing effects from α-induced decreases in S_out_ and LAI-driven increases in LE found mainly in southern China (Fig. 4g, j; Supplementary Figs 7b, c and 8b, c). Interestingly, winter L_in_ is found to increase (Fig. 4j), driven mainly by the higher values of near surface water vapor content over the coastal areas of southern China (Supplementary Fig. 8g). This excess of surface water vapor is brought further to the north by the changed circulation, resulting in an increase in column-integrated precipitable water and L_in_ over the regions east of the central Yangtze River basin (Supplementary Fig. 8d, h).

### Teleconnections between vegetation changes in China and T_a_ response in other regions

**Figure 5.**
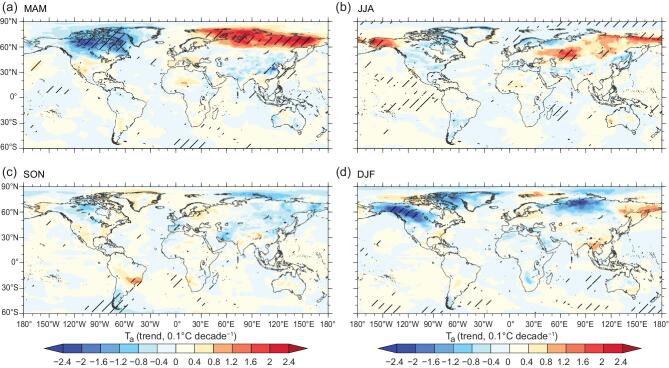
Trend in global surface air temperature in response to afforestation and vegetation greening in China from 1982 to 2011. The trend of the air temperature (T_a_) was computed for SCE minus CTL (see Methods) in boreal (a) spring (March, April and May, MAM); (b) summer (June, July and August, JJA); (c) autumn (September, October and November, SON) and (d) winter (December, January and February, DJF). Hatching indicates a 95% confidence level.

We further quantify how T_a_ over the rest of the globe may respond to vegetation changes restricted to China by our experimental design. Afforestation and vegetation greening in China exert effects on T_a_ in other regions through reorganization of the atmospheric general circulation. We find that China’s vegetation greening has the largest teleconnected impact on spring T_a_ over the northern high latitudes. Vegetation changes in China cause a dipole effect, with warming (0.17°C decade^−1^) over the Russian Arctic (66.5 − 90^o^N, 30°E−180) and cooling (−0.13°C decade^−1^) over the Canadian Arctic (66.5 − 90^o^N, 140 − 60^o^W) (Fig. 5a). The vegetation changes in China account for ∼58% of the simulated spring warming over the Russian Arctic and ∼61% of the cooling over the Canadian Arctic. Generally, regions outside China are impacted insignificantly during the summer, although a remote warming effect is induced over Kazakhstan, eastern Mongolia and Alaska (Fig. 5b). There is no obvious T_a_ response in autumn, but there is a significant decrease in T_a_ over the northwest of North America in winter (Fig. 5c, d).

**Figure 6.**
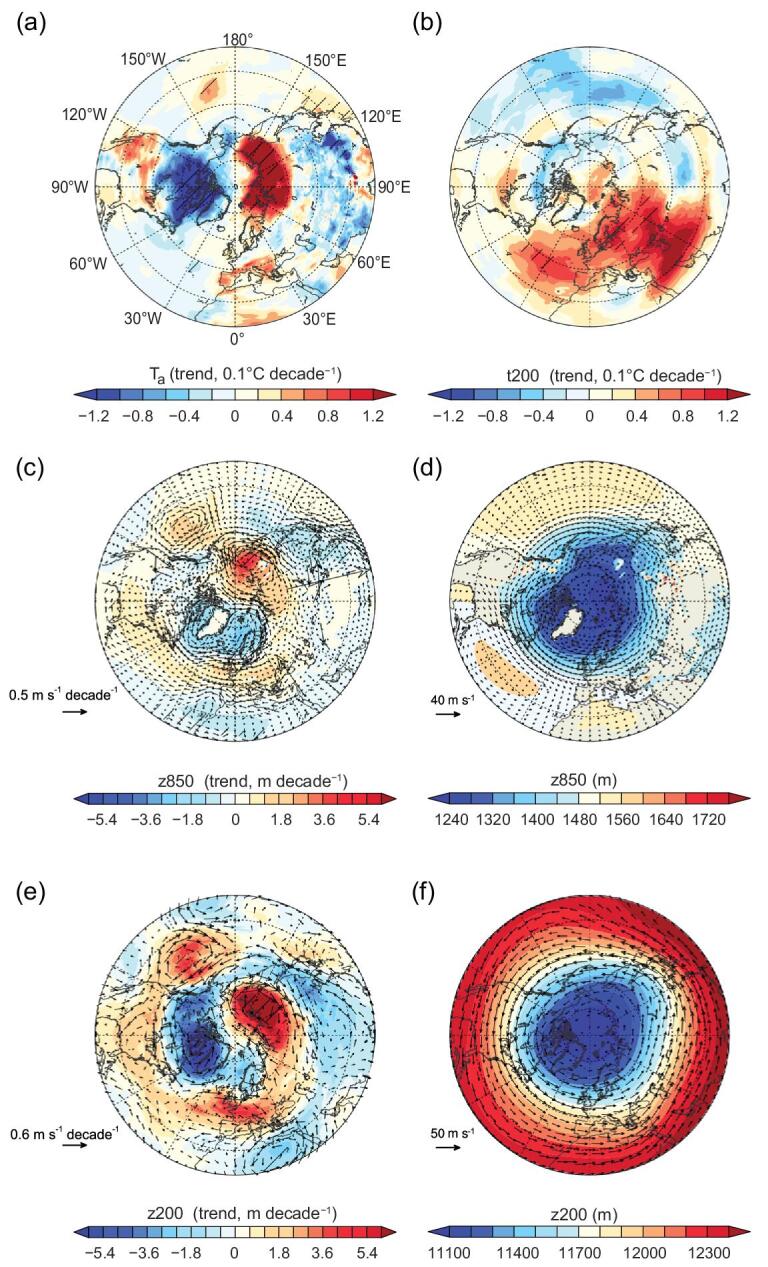
Spring trend in air temperature and geopotential height in response to afforestation and vegetation greening in China from 1982 to 2011. Trend in air temperature at (a) surface (T_a_), (b) 200 hPa (t200) geopotential height, (c) 850 hPa (z850) and (e) 200 hPa (z200), computed for SCE minus CTL (see Methods) from 1982 to 2011. Climatological (CTL) geopotential height is shown in (d) 850 hPa and (f) 200 hPa. The trend in wind vector and its climatology are overlaid on (c–f) at the corresponding pressure level. For the computed trend, the hatching indicates a 95% confidence level.

There are substantial changes of the atmospheric circulation in the Northern Hemisphere, especially during the spring (Fig. 6). Surface cooling, driven by ET enhancement, in North China produces a negative trend of geopotential height at 850 hPa in East Asia (Fig. 6c, d). There is a wave train propagating to the Arctic with a positive trend over the Bering Sea and a negative trend over the Greenland Sea. This wave train structure is enhanced at 200 hPa (Fig. 6e), with an upstream shift of the two high-latitude anomalous trend centers. The trend in 200 hPa geopotential height shows a strong spiral structure, certainly revealing variations in the atmospheric jets that spiral at this altitude. The wave train entering the Arctic region may partly break up the Arctic vortex (Fig. 6f) and cause large-amplitude upper-level and surface air temperature variations in the regions around the Arctic Ocean (Fig. 6a, b).

To verify the robustness of the Arctic tropospheric response to spring greening over China, we analyze the results of each member of the paired SCE and CTL simulations. It is found that there are 12 out of 30 members that produce a similar spatial pattern of teleconnections between vegetation changes in China and the Arctic climate, with the trend of geopotential height in the upper-level troposphere over the Northern Hemisphere illustrated in Fig. 6e. For the other three seasons, there is a large difference in the trend of 850 and 200 hPa geopotential height between individual members (Supplementary Figs 9–11).

## DISCUSSION

The IPSLCM is able to capture the observed interannual variation of T_a_ over China (Fig. 1) when driven by increasing atmospheric CO_2_ concentration and observed SST from 1982 to 2011. Both the observations and simulation show a warming slowdown, in particular after the year 2000 during the winter (Supplementary Fig. 1a−d). This phenomenon is part of the global warming hiatus, which has been attributed to meridional energy transport via the ‘atmospheric bridge’ originating in surface cooling of the tropical central and eastern Pacific Ocean [40]. Prescribed with observed global ocean SST, our SCE simulations can capture the observed decrease of winter T_a_ across China for 2002–2011 (Supplementary Fig. 12 versus Fig. 2 in Kosaka and Xie [40]), confirming that ocean SST plays a critical role in the winter warming hiatus over China [41]. In addition, the model’s inability to reproduce accurately the interannual amplitude of the winter T_a_ (Supplementary Fig. 1b) can be attributed to underestimation of the variability of the East Asian winter monsoon [42] by the multi-member ensemble average, as evidenced by the underestimated amplitude of the Siberian High (Supplementary Fig. 1i). In contrast to the overall warming, driven by increasing CO_2_ and SST, local cooling is observed in the Sichuan Basin and in parts of Northwest, North and Northeast China (Fig. 1a). Temperature decreases over these regions might be explained by changes in other factors, such as increasing aerosol optical depth (AOD) and expansion of irrigation schemes. For instance, the prominent cooling over the Sichuan Basin in Southwest China is probably a result of increased AOD that reduces local solar radiation [43]. Our simulations did not capture these local cooling effects because the experiment design only considers variations in CO_2_, SST, LAI and forest cover. We highlight the necessity to consider other processes (related to aerosols, in particular) in future investigations.

**Figure 7.**
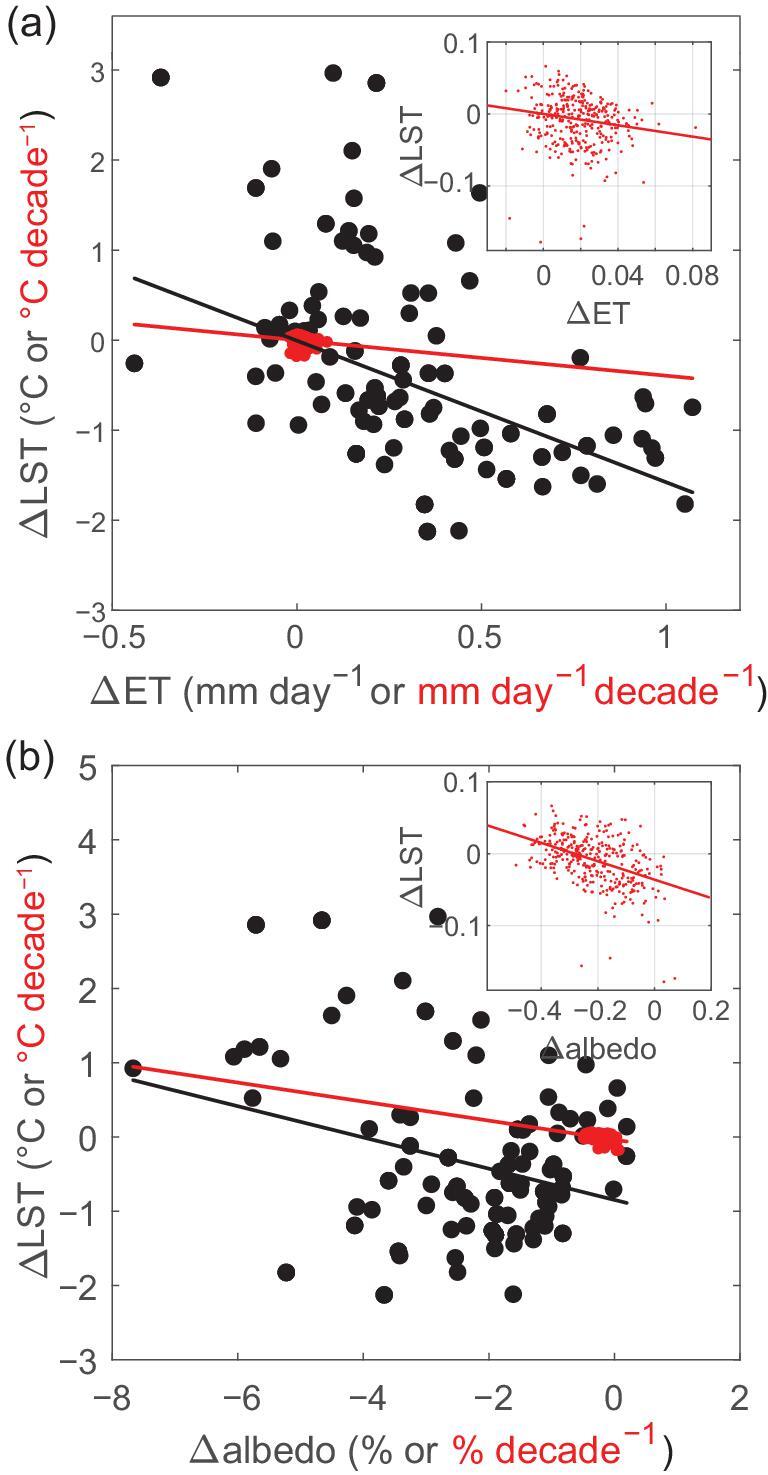
Scatter plots of vegetation-induced change in land surface temperature (LST) as a function of evapotranspiration (ET) (a) and albedo (b). Plots are shown for both the observed spatial relationship (black dots) and for the simulated temporal trend (red dots). Observed data were randomly sampled to generate 1000 grid cells that have at least 10% fractional cover of planted forest, and these then were filtered by eliminating those with an elevation difference exceeding the range −100∼100 m, as described in Peng *et al*. [8]. The threshold of 10% forest fraction over a 40 × 40 km grid cell is approximately equal to 1.85% in our model grid cells (∼120 × 72 km). Therefore, model grid cells with a rate of increased forest cover of at least 1.85% decade^−1^ are shown as red dots on the plot. For the satellite-observed data, LST was computed as the average between daytime and nighttime LST, while for the model output it was calculated from the upward longwave radiation using the Stefan-Boltzmann law.

Surface biophysical effects are shown to dominate the annual T_a_ response to afforestation and vegetation greening over China, evidenced by the vegetation-induced reduction of S_out_ by decreased α, mainly in southern China and the enhancement of surface ET across the whole country (Figs 2c, d and 3a, b, c, h, i; Supplementary Fig. 4a, i). Decreased α implies increased absorbed solar incoming radiation, potentially leading to increased surface temperature, in particular over needle-leaf forest in snow-covered high latitudes [44,45]. Significant enhancement of ET compensates for lower α in energy-limited forests (e.g. in wet sub-tropical and tropical regions) [14]. As shown in a previous study [8], replacing short vegetation with planted forests tends to have higher ET and lower α that cause lower and higher LST, respectively (see black dots in Fig. 7). Our model simulations reproduce the observed negative correlation between LST and α (ET) over grid cells where afforestation occurs (see red dots in Fig. 7), although the magnitude is smaller than observed. In contrast to previous studies that usually assume a complete replacement (i.e. 100%) of grassland with forest for the purpose of enhancing the signal of complete land-use transitions [8,46], here the average increase of forest cover in China is merely 3.63 ± 1.37% decade^−1^. A mean decrease in daily LST of −0.26°C for full afforestation [8] would be dampened to −0.009 ± 0.0036°C decade^−1^ in a pixel with this smaller observed rate of afforestation (Supplementary Fig. 13). The simulations indicate that realistic increases in forest area and LAI (the observed rates from 1982 to 2011 were used in this study) would lead only to small changes in surface ET and α, resulting in a correspondingly small change in the surface climate (Fig. 7). Thus, our results imply that, from the biophysical point of view, afforestation and vegetation greening in China have not yet reached a scale large enough to cause major temperature effects.

In contrast to the impacts of change in α and ET on the surface energy balance, decreased r_a_ has been found to exert limited effects on Hs (Figs 2c, d and 3l). This is due to the simultaneous decline of T_s_ − T_a_ in Equation (4) as shown in Methods. The widespread decrease in r_a_, on average − 0.32 ± 0.05 s m^−1^ decade^−1^ (*P* < 0.05) (Supplementary Fig. 4f), is due to the increase in surface roughness, which is expected to increase local turbulent flow and heat exchange with the near-surface air [47]. Thus, the climate effect of the change in roughness is an important biophysical mechanism that was initially quantified through a decomposition of surface temperature into different energy exchange processes [48]. Compared to the temperature decomposition described in Lee *et al*. [48], a key difference here is that we do not combine the two turbulent fluxes LE and Hs by using the concept of the Bowen ratio (Equation 9 versus Equation 12 in Methods). Instead, as detailed in Zeng *et al*. [28], the vegetation-induced change in ET is regarded as an independent term in our decomposition. A further derivation (see Methods) reveals that the two decomposition methods are two simplified forms of the feedback loop between the surface and atmosphere. We can demonstrate that the cooling effects of ET enhancement would be attributable to effects caused by the change in surface roughness if LE and Hs were integrated following Lee *et al.* [48] (Supplementary Fig. 14). Although roughness-induced change in r_a_ impacts ET in land surface models [49], clarifying this conceptual difference could help us focus on the source of uncertainties in more complex atmospheric and hydrological processes (e.g. vapor pressure deficit, temperature and leaf water potential) rather than on factors that only affect the simulated roughness. Nonetheless, further evaluations of the process of model-simulated roughness and r_a_ also are critical, because their potential simulation bias for different vegetation types may influence the simulated coupling strength between vegetation and atmosphere [50], contributing to a major uncertainty in projecting the ecosystem-scale transpiration response to increasing CO_2_ in the future [51]. Although included in our results, vegetation physiological response to increasing CO_2_ and its impacts on plant water use efficiency and surface air temperature are expected to be isolated further from the integrated effects of the increased vegetation cover in China using additional simulations (e.g. fixing CO_2_ concentration at a constant level). This future work could be particularly useful due to the increased importance of vegetation physiology for reducing terrestrial ET and increasing air temperature under various scenarios, such as doubled atmospheric CO_2_ concentration [38], for example.

Vegetation-induced increase in L_in_ over Southeast China and the reduction in S_in_ over the Yangtze River basin (Figs 2c, d and 3g, j) indicate the importance of atmospheric feedback on the surface energy balance [12,13]. By modifying the surface moisture supply and thermal properties, vegetation is able to alter atmospheric water vapor transport, in particular that associated with change in large-scale circulations (e.g. the Intertropical Convergence Zone and Asian monsoons) [52–55]. Previously, we have shown that realistic vegetation cover changes in China would result in an overall weak impact on the East Asian summer monsoon and that the vegetation regulation of low-level wind could alter regional precipitation response [32]. We also find that the regulation of low-level wind by vegetation could change the spatial distribution of increased water vapor stemming from afforestation and vegetation greening. For example, due to the significant enhancement of LE, Q2m significantly increases during the spring season over both Southeast and North China (Supplementary Fig. 5c, g). However, the vegetation-induced trend in the southwesterly wind increases the water vapor in southern China and adjacent seas, resulting in an increased L_in_ in Southeast China (Supplementary Fig. 5d, h). Note that it has been suggested that the potential spring irrigation in India would advect more water vapor to southern China [56]; L_in_ could thus increase more over Southeast China, and the combined increase in spring water vapor would be advected further to Southeast Asia and to the Pacific. Although not considered in our model, irrigation expansion (expansion of the area irrigated and, in some regions, the amount of irrigation water applied) has been reported over cropping areas in Northwest, North and Northeast China over the past decades [57]; this also may contribute to spring LE enhancement, for example, as well as increasing water vapor content in northern China.

The largest vegetation-induced cooling effect over China is found during the spring. The key mechanism here is the LAI-driven enhancement in surface LE (Fig. 4; Supplementary Fig. 5) under, mostly, energy-limited ET regimes (Supplementary Fig. 15d). This LAI control on the spatial pattern of changes in LE implies a high value of the ratio of vegetation transpiration to terrestrial ET (T/ET) of about 62.6% in this study. This value is in agreement with the global T/ET value found in the IPSLCM, but higher than the values from most other models participating in the Coupled Model Intercomparison Project Phase 5 (CMIP5) [58]. Evidence has accumulated that model-simulated T/ET may be related to T_a_ response to vegetation cover changes. For example, by comparing results from three coupled models at the global scale, Zeng *et al.* [28] found that the models with a higher value of T/ET, such as the IPSLCM, tend to simulate a higher sensitivity of T_a_ to increased LAI, indicating the key role of T/ET for the coupling strength between vegetation cover and T_a_.

Notably, our finding that the largest cooling effect is in the spring is different from the conclusions of previous studies that have suggested that the highest ET-induced cooling in temperate forests occurs during the summer [21,59,60]. Such seasonal divergence is caused by differences in the seasonality of changes in LAI. The assumption in previous studies of complete replacement of grass with forest would imply that the largest change in LAI occurred during the summer [61]. In contrast, the seasonality of the trend in satellite-observed LAI may vary, depending on CO_2_ fertilization, climate change, nitrogen deposition and land-use change [31,62]. In particular, vegetation growth is shown to be suppressed by the increased warming and, therefore, the trend in summer LAI is smaller than that in the spring over China, as shown by data from the Global Inventory Modeling and Mapping Studies (GIMMS) in Zhu *et al*. [63]. The observed spring greening and its widespread cooling effects shown in this study would thus feedback negatively to the advance of the green-up date [64,65], because T_a_ is a primary factor determining the spring phenology of temperate vegetation over China [66].

Further computations of extreme temperature indices [67] based on daily output indicate that spring vegetation greening efficiently would reduce warm (daily max temperature > 90th percentile) and summer (daily max temperature > 25°C) days, mainly in North China and the Yangtze River basin (Supplementary Fig. 16a, c). In contrast, South China may experience a risk of increased warm days (Supplementary Fig. 16 g, m, s) due to a lack of sufficient latent heat flux to partition the surplus shortwave radiation resulting from reduced α in the other three seasons (Supplementary Figs 6b, c, 7b, c and 8b, c). Our results thus highlight the contrasting biophysical effects on extreme temperatures between different seasons, which need to be considered in the evaluation of future large-scale afforestation projects.

In addition to local cooling effects, spring greening was found to cause a decrease in geopotential height, both in the upper and lower troposphere over China, which exerts a significant influence on T_a_ in the Arctic via atmospheric teleconnections (Figs 5a and 6). As the geopotential height decreases over China, the upper-level meridional temperature and pressure gradients between the Arctic and China are reduced proportionately, resulting in a reduction in the strength of the westerlies over Mongolia (Fig. 6e). Because of the global linkage between the Arctic troposphere and the prevailing westerlies [68], this regional wind perturbation could contribute to the slowing of the eastward progression of Rossby waves and, hence, to an increased likelihood of more persistent weather systems [69]. Further studies on the teleconnected influence on spring extreme events and the seasonal transition in the Arctic terrestrial ecosystem are greatly needed. Because the atmospheric contributions from other regions are likely to augment or compensate for the remote effects of Chinese vegetation as revealed in this study, it is equally important to analyze the atmospheric feedback from the land and ocean surface in regions outside China.

It also is worth noting that the high-latitude response of T_a_ and geopotential height to spring greening in China is to some degree consistent with previous simulations by Hoskins and Karoly [70], who found that the height field perturbation in high latitudes mainly occurs in response to midlatitude thermal forcing. Hoskins and Karoly [70] regard the subtropical-midlatitude forcing as a source of Rossby waves that could propagate poleward to affect the climate at high latitudes. This mechanism has been used to explain the simulated teleconnections between Amazonian deforestation and changes in the Northern Hemisphere climate [71], although the robustness of the signal is determined partly by model resolution [72]. Whether the teleconnection found here would be dampened or amplified if the spatial resolution of the IPSLCM was improved further remains an open question.

In summary, we used a coupled land-atmosphere global climate model to investigate surface and atmospheric biophysical climate effects from large-scale afforestation and vegetation greening over China during 1982–2011. The model shows that the observed increase in forest area and LAI resulting from the world’s largest afforestation project would cause regionally contrasting temperature effects with northern cooling and southern warming in China due to both small and counteracting effects between decreased α and enhanced ET. Despite such observable surface biophysical effects, we provide model evidence that the spatial pattern of the surface energy balance could be strongly affected by atmospheric biophysical effects, such as vegetation-induced changes in cloud cover and atmospheric water vapor content. These latter two further affect the vegetation-induced trends in surface downward shortwave and longwave radiation. The strongest cooling effects caused by enhanced ET are found during the spring when the greening over China is simulated to cause a robust decrease in regional geopotential height and produce a ‘China Low-Arctic Dipole’ structure in the trend of upper-level geopotential height. This vegetation-induced structure may partly account for spring warming over the Arctic regions of Russia and spring cooling in Canada. Future studies need to focus on verifying the robustness of these findings with more models and exploring the potential climate consequences of spring greening over China on the Arctic ecosystem.

## METHODS

### Coupled land-atmosphere global climate model zoomed over China

The climate model used in this study is the Institute Pierre Simon Laplace climate model (IPSLCM), which has widely participated widely in multiple model intercomparison projects, such as the Coupled Model Intercomparison Project Phase 5 (CMIP5) [30]. The model configuration used here consists of the atmospheric model, LMDz (the Laboratoire de Météorologie Dynamique atmospheric general circulation model with zoom capability), which represents the fundamental dynamical and physical processes in the atmosphere [73] and the land surface model, ORCHIDEE (ORganizing Carbon and Hydrology In Dynamics EcosystEms), which represents vegetation dynamics and interactive carbon cycle in the terrestrial ecosystem [74]. The other components of the IPSLCM relating to ocean dynamics and sea-ice were not activated in this study, but instead were replaced by prescribing the observed sea-surface temperature (SST) and sea-ice (SIC) as lower boundary conditions. The model has 19 vertical levels with an irregular horizontal grid, which was zoomed in inside the longitudinal and latitudinal range of China (central spatial resolution: ∼1^o^ × 0.6^o^) while being coarser over regions outside China (spatial resolution: ∼4^o^ × 2^o^). This grid design ensures a free exchange of atmospheric energy, water and momentum between the inside and outside of the zoomed region and avoids the potential effects of fixed boundary climate forcing that dampens vegetation biophysical feedback to the atmospheric general circulation.

Except for bare soil, the ORCHIDEE model divided the terrestrial vegetation into 12 plant functional types (PFTs, http://forge.ipsl.jussieu.fr/orchidee/wiki/VegetMap), with each matched with an independent vegetation canopy and physiological parameters to calculate the vegetation-related physical and biological processes. For the simulation over China, physiological parameters in five of these PFTs (i.e. tropical evergreen broadleaf forest, temperate evergreen broadleaf forest, temperate evergreen needle-leaf forest, temperate deciduous broadleaf forest and boreal deciduous needle-leaf forest) were previously optimized according to observed carbon and water fluxes from six forest eddy-covariance sites in China [75]. Vegetation canopy parameters, such as the leaf area index (LAI) and forest cover, were derived from satellite observations obtained from the Global Inventory Modeling and Mapping Studies (GIMMS) [76] and the reconstructed forest cover map from the inventory investigation [3] (see supporting information in Li *et al*. [32]). The SST and SIC map was derived from the Atmospheric Model Intercomparison Project (AMIP; www.pcmdi.llnl.gov/projects/amip) at a 1-degree spatial resolution, and observed global atmospheric CO_2_ data were obtained from those used for transient modeling in the project ‘Trends in net land-atmosphere carbon exchange’ (TRENDY, http://dgvm.ceh.ac.uk/node/9) for 1982–2011.

### Model experiments and evaluation of simulated results

Two 30-year transient simulations were performed using the IPSLCM (LMDz/ORCHIDEE) coupled model: (i) The dynamic vegetation scenario (SCE) experiment was forced with observed LAI, forest cover map, sea surface temperature (SST) and CO_2_ concentration during the historical time period 1982–2011 and, (ii) in the control (CTL) experiment, the lower-boundary conditions (SST and SIC) and radiative forcing (CO_2_) are identical to SCE, but LAI prescribed by a seasonal climatology and forest cover in each grid cell within China remains static, as of the year 1982, throughout the experimental period 1982—2011. This experiment builds on Li *et al*. [32] by extending the original 15-member ensemble into a more robust 30-member ensemble, with each member in SCE corresponding to a member in CTL that has the same initial atmospheric conditions. The ensemble mean was used in the analysis. The experiment design resembles that in AMIP and has been demonstrated to reasonably simulate the temporal variations in surface air temperature (T_a_) at the global scale [28] and in China, as shown by this study. The trends in the SCE minus CTL T_a_ and surface energy balance were computed and viewed as the climate effects from vegetation cover changes in China over the past 30 years.

To further demonstrate the model’s capability in simulating T_a_ and energy fluxes in China, we used observations or observation-based data from multiple sources to evaluate the model simulations. Observed T_a_ was derived from the CRU TS3.21 data sets [33], while the observed radiation fluxes (including upward and downward shortwave and longwave radiation) were obtained from Clouds and the Earth’s Radiant Energy System (CERES) SYN1deg-Month [34]. Radiation fluxes were obtained from two reanalysis data sets. The European Centre for Medium-Range Weather Forecasts (ECMWF) reanalysis (ERA-Interim) [35] and National Centers for Environmental Prediction (NCEP) [36], were also used. The observation-based ET products were derived from Jung *et al*. [39] and Zeng *et al*. [77].

### Decomposition of vegetation-induced trend in T_a_

We applied the decomposition method from Zeng *et al*. [28] to better assess processes and mechanisms involved in vegetation biophysical effects. This decomposition was based on the fundamental equation of the surface energy balance:

(1) \begin{equation*} {S}_\mathit{in}-{S}_\mathit{out}+{L}_\mathit{in}-{L}_\mathit{out}= \mathit{LE}+ \mathit{Hs}+G \end{equation*}

where S_in_ and S_out_ represent surface downward and upward shortwave radiation, respectively. L_in_ and L_out_ represent surface downward and upward longwave radiation, respectively. LE, Hs and G denote the latent heat flux, sensible heat flux and the ground flux, respectively. Specifically, ${S}_\mathit{in}-{S}_\mathit{out}$, ${L}_\mathit{in}-{L}_\mathit{out}$ and $Hs$ are given by:

(2) \begin{equation*} {S}_\mathit{in}-{S}_\mathit{out}={S}_\mathit{in}\left(1-\alpha \right) \end{equation*}

(3) \begin{equation*} {L}_\mathit{in}-{L}_\mathit{out}={\varepsilon}_s\sigma \left({\varepsilon}_a{T}_a^4-{T}_s^4\right) \end{equation*}

(4) \begin{equation*} Hs=\rho {C}_p\frac{T_s-{T}_a}{r_a} \end{equation*}

where $\alpha$, ${\varepsilon}_s$, $\sigma$, ${\varepsilon}_a$, ${T}_a$, ${T}_s$, $\rho$, ${C}_p$ and ${r}_a$ denote surface shortwave albedo, land surface emissivity, Stephan–Boltzmann constant (5.67 × 10^−8^ W m^−2^ K^−4^), atmospheric air emissivity, land surface 2-m air temperature, land surface temperature, air density (1.21 kg m^−3^), specific heat capacity of air at constant pressure and the aerodynamic resistance, respectively.

Using Equations (2)–(4) and differentiating Equation (1) with respect to T_s_, we can obtain an expression for $\Delta {T}_s$ (see detailed derivation processes in Zeng *et al*. [28]):

(5) \begin{eqnarray*} &&\Delta {T}_s = \frac{1}{f_s}\Big(-{S}_\mathit{in}\Delta \alpha +\Delta {S}_\mathit{in}\left(1-\alpha \right)-\Delta LE\\ &&+\,\frac{\rho {C}_p}{r_a^2}\left({T}_s-{T}_a\right)\Delta {r}_a+{\varepsilon}_s\sigma {T}_a^4\Delta {\varepsilon}_a\Big)\nonumber\\ &&+\left(\frac{\rho {C}_p}{r_a}+4{\varepsilon}_s{\sigma \varepsilon}_a{T}_a^3\right)\Delta {T}_a \Big/\nonumber\\ &&\quad\left(\frac{\rho {C}_p}{r_a}+4{\varepsilon}_s\sigma {T}_s^3\right) \end{eqnarray*}

where ${f}_s$ is the energy redistribution factor, given by:

(6) \begin{equation*} {f}_s=\frac{\rho {C}_p}{r_a}+4{\varepsilon}_s\sigma {T}_s^3 \end{equation*}

By assuming a conversion coefficient in front of the first term (i.e. ${T}_s^\mathit{rad}=\frac{1}{f_s}(-{S}_\mathit{in}\Delta \alpha +\Delta {S}_\mathit{in}(1-\alpha )-\Delta \mathit{LE}+\frac{\rho {C}_p}{r_a^2}({T}_s-{T}_a)\Delta {r}_a+{\varepsilon}_s\sigma {T}_a^4\Delta {\varepsilon}_a)$) on the right-hand side of Equation (5) to represent the transformation of the surface radiative flux to T_a_:

(7) \begin{eqnarray*} {T}_a^\mathit{rad}&=&\ \left(\left(\rho {C}_p/{r}_a+4{\varepsilon}_s\sigma {T}_s^3\right)\big/\right.\nonumber\\ &&\left.\left(\rho {C}_p/{r}_a+4{\varepsilon}_s{\sigma \varepsilon}_a{T}_a^3\right)\right){T}_s^\mathit{rad} \end{eqnarray*}

Zeng *et al.* [28] obtained the equation for decomposing T_a_ into separate factors:

(8) \begin{eqnarray*} &&\Delta {T}_a &=&\ \frac{1}{f}\Big(-{S}_\mathit{in}\Delta \alpha +\Delta {S}_\mathit{in}\left(1-\alpha \right)-\lambda \Delta E\nonumber\\ &&\quad+\frac{\rho {C}_p}{r_a^2}\left({T}_s-{T}_a\right)\Delta {r}_a+{\varepsilon}_s\sigma {T}_a^4\Delta {\varepsilon}_a\Big)\nonumber\\ \quad+\Delta {T}_a^\mathit{cir} \end{eqnarray*}

where $f$ is the energy redistribution factor for T_a_, given by:

(9) \begin{equation*} f=\frac{\rho {C}_p}{r_a}+4{\varepsilon}_s{\sigma \varepsilon}_a{T}_a^3 \end{equation*}

$-{S}_{in}\Delta \alpha$, $-\lambda \Delta E$, $\frac{\rho {C}_p}{r_a^2}({T}_s-{T}_a)\Delta {r}_a$, $\Delta {S}_{in}(1-\alpha)$ and ${\varepsilon}_s\sigma {T}_a^4\Delta {\varepsilon}_a$ represent the climate forcing and response induced by vegetation cover changes through altering surface albedo (α), evapotranspiration (ET), aerodynamic resistance (r_a_), shortwave (SW) radiation and air longwave radiative emissivity (${\varepsilon}_a$), respectively. The sum of these terms denotes the combined forcing and response in Fig. 2. Although the assumption of the conversion coefficient in front of the radiative part of ${T}_s$ in Equation (7) lacks a rigorous physical derivation, it can be said to represent the climate forcing and response generated by vegetation cover changes on T_a_, at least representing the vegetation-induced long-term trend in T_a_ and energy fluxes [28].

### Conceptual variants on the intrinsic biophysical mechanisms analysis

Equation (8) shows that our decomposition method regards the vegetation-induced change in ET as an independent term, which is different from the well-known derivation of the vegetation-induced change in surface energy balance from Lee *et al*. [48]:

(10) \begin{equation*} \Delta {T}_s\approx \frac{\lambda_0}{1+{f}^{\prime }}\Delta {S}_n+\frac{-{\lambda}_0}{{\left(1+{f}^{\prime}\right)}^2}{R}_n\left(\Delta {f}_1+\Delta {f}_2\right)\end{equation*}

where ${\lambda}_0$, ${f}^{\prime }$, $\Delta {f}_1$ and $\Delta {f}_2$ are given by:

(11) \begin{equation*} {\lambda}_0=\frac{1}{4\sigma {T}_a^3}\end{equation*}

(12) \begin{equation*} {f}^{\prime }=\frac{\rho {C}_p}{4\sigma {T}_a^3{r}_a}\left(1+\frac{1}{\beta}\right)\end{equation*}

(13) \begin{equation*} \Delta {f}_1=\left(1+\frac{1}{\beta}\right)\frac{\rho {C}_p}{4\sigma {T}_a^3}\left(-\frac{\Delta {r}_a}{r_a^2}\right) \end{equation*}

(14) \begin{equation*} \Delta {f}_2=\frac{\rho {C}_p}{4\sigma {T}_a^3{r}_a}\left(-\frac{\Delta \beta }{\beta^2}\right) \end{equation*}

${R}_n$ and $\beta$ denote surface net radiation and Bowen ratio, respectively. It has been suggested that the first term in Equation (10) indicates the vegetation forcing through the radiative process, while $\Delta {f}_1$ and $\Delta {f}_2$ indicate the vegetation biophysical impacts on climate through altering surface roughness and the Bowen ratio. The contribution of vegetation roughness often is claimed to be the largest part, deduced from some *in situ* measurements [12,48].

To clarify the conceptual variants for distinguishing the relative importance of vegetation-induced change in surface roughness or ET, we used Equation (S7) from the supplementary data in Lee *et al.* [48] but considered LE as a primary variable rather than a secondary variable behind the Bowen ratio $\mathit{LE}= \mathit{Hs}/\beta$:

(15) \begin{equation*} {T}_s-{T}_a=\frac{\lambda_0}{1+{f}^{{\prime\prime} }}\left({R}_n^{\ast }- LE-G\right) \end{equation*}

${f}^{{\prime\prime}}$ is given by:

(16) \begin{equation*} {f}^{{\prime\prime} }=\frac{\rho {C}_p}{4\sigma {T}_a^3{r}_a} \end{equation*}

Then, we can obtain a new equation:

(17) \begin{eqnarray*} \Delta {T}_s &\approx&\ \frac{\lambda_0}{1+{f}^{{\prime\prime} }}\Delta {S}_n-\frac{\lambda_0}{1+{f}^{{\prime\prime} }}\Delta LE\nonumber\\ &&+\,\displaystyle\frac{-{\lambda}_0}{{\left(1+{f}^{{\prime\prime}}\right)}^2}\left({R}_n- LE\right)\Delta {f}^{\prime}\end{eqnarray*}

(18) \begin{equation*}{\Delta} {f}^{\prime }=\frac{\rho {C}_p}{4\sigma {T}_a^3}\left(-\frac{\Delta {r}_a}{r_a^2}\right) \end{equation*}

where the terms related to $\Delta LE$ and $\Delta {f}^{\prime }$ indicate the contribution of vegetation climate forcing from surface ET and r_a_, respectively.

This modified method and the method given in Lee *et al*. [48] were both applied to decomposing the model-simulated change in T_a_ induced by vegetation cover changes. Because the two methods used the same data, the results (Supplementary Fig. 14) should reveal any conceptual variants between the different decomposition methods. With this approach, we can conclude that vegetation-induced change in ET dominated by the observed LAI is the main influencing mechanism on the seasonality of vegetation biophysical feedback.

## Supplementary Material

nwz132_Supplemental_File
