# Supplementary material for: Local and teleconnected temperature effects of afforestation and vegetation greening in China
Source: Natl Sci Rev. 2019 Sep 12;7(5):897–912. doi: 10.1093/nsr/nwz132 (PMC8289082; doi:10.1093/nsr/nwz132)

**Supplementary Information for**

**Local and tele-connected temperature effects of afforestation and vegetation greening in China**

Yue Li^1^, Shilong Piao^1,2,3,*^, Anping Chen^4^, Philippe Ciais^5^, Laurent Z. X. Li^6^

^1^Sino-French Institute for Earth System Science, College of Urban and Environmental Sciences, Peking University, Beijing 100871, China.

^2^Key Laboratory of Alpine Ecology and Biodiversity, Institute of Tibetan Plateau Research, Chinese Academy of Sciences, Beijing 100085, China.

^3^Center for Excellence in Tibetan Earth Science, Chinese Academy of Sciences, Beijing 100085, China.

^4^ Department of Biology, Colorado State University, Fort Collins, CO 80523, USA.

^5^Laboratoire des Sciences du Climat et de l’Environnement/Institut Pierre Simon Laplace, Commissariat à l’Énergie Atomique et aux Énergies Alternatives–CNRS–Université de Versailles Saint-Quentin, Université Paris-Saclay, F-91191 Gif-sur-Yvette, France.

^6^Laboratoire de Météorologie Dynamique, Centre National de la Recherche Scientifique, Sorbonne Université, Ecole Normale Supérieure, Ecole Polytechnique, 75252 Paris, France.

**Supplementary Table 1. Evaluations of the surface energy fluxes on the simulated trend and correlation coefficient with observations.** Trend and correlation coefficient (R) are computed for downward (S_in_), upward (S_out_), net (S_net_) shortwave radiation, downward (L_in_), upward (L_out_), net (L_net_) longwave radiation, surface net radiation (R_n_) and latent heat flux (LE). Observed radiation fluxes are derived from the Clouds and the Earth’s Radiant Energy System (CERES) [34] and the latent heat flux is derived from observation-based dataset from Ref. [39]. To keep consistency with the observed datasets, radiation fluxes and latent heat flux from SCE are computed during the time period 2001−2011 and 1982−2011, respectively.

| **Trend**  (W m^−2^/10 yr) | **S_in_** | **S_out_** | **S_net_** | **L_in_** | **L_out_** | **L_net_** | **R_n_** | **LE** |
| --- | --- | --- | --- | --- | --- | --- | --- | --- |
| CERES | 2.33 | 0.87 | 1.46 | −**4.26*** | −1.59 | −2.67 | −1.21 | ― |
| Jung et al. | ― | ― | ― | ― | ― | ― | ― | **0.44*** |
| SCE | −**5.13*** | −**1.14*** | −**3.99*** | **1.83*** | −**1.26*** | 3.09 | −0.90 | 0.15 |
| R_SCE&Obs | **0.74*** | 0.26 | **0.67*** | 0.34 | 0.34 | 0.11 | 0.53 | **0.43*** |

* *P* < 0.05, R indicates correlation coefficient computed by detrended energy fluxes between SCE simulations and the observations.

**Supplementary Fig. 1 Seasonal difference in observed and simulated warming in China from 1982 to 2011. a**, **b**, **c**, **d**, 30-year temporal variations of air temperature (T_a_) anomaly (minus 30-year mean) based on observed dataset from the Climate Research Unit (CRU TS3.21) [33] and SCE simulation. **e**, **f**, **g**, **h**, scatter plot of observed and simulated T_a_ anomaly. T_a_ have been averaged for summer (June, July and August, JJA, panels **a** and **e**), winter (December, January and February, DJF, panels **b** and **f**), spring (March, April and May, MAM, panels **c** and **g**) and autumn (September, October and November, SON, panels **d** and **h**). **i**, the anomaly of winter Siberian High, computed by the sea level pressure averaged from three nearest grid cells to 60^o^N, 100^o^E; 60^o^N, 90^o^E; 50^o^N, 100^o^E [42]. The sea level pressure is subtracted by 30-year mean with the sign reversed to represent the impact on winter temperature. Light grey line denotes computed anomaly of winter Siberian High from each member in SCE. Magenta and red lines denote the data computed from reanalysis datasets.

**
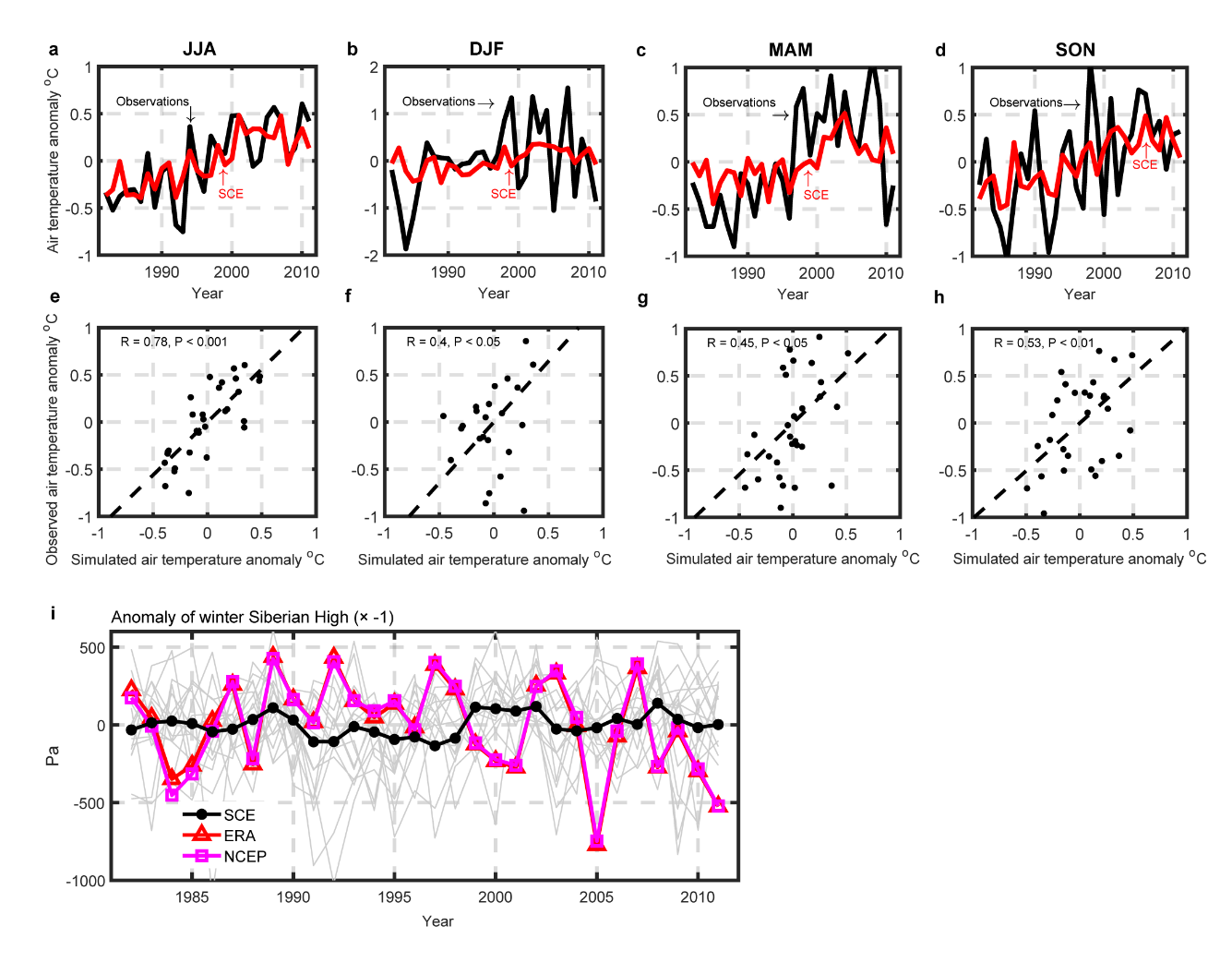
**

**Supplementary Fig. 2 The spatial patterns of climatological downward shortwave and longwave radiation from model and satellite observation.** The climatological downward shortwave (S_in_) and longwave radiation (L_in_) are averaged over 2001−2011 when the satellite-observed data are available from Clouds and the Earth’s Radiant Energy System (CERES) [34]. Model simulations from SCE experiment are also shown to be compared to the observed data at the annual time scale (ANN) and during the spring (March, April and May, MAM), summer (June, July and August, JJA), autumn (September, October and November, SON) and winter season (December, January and February, DJF).

**
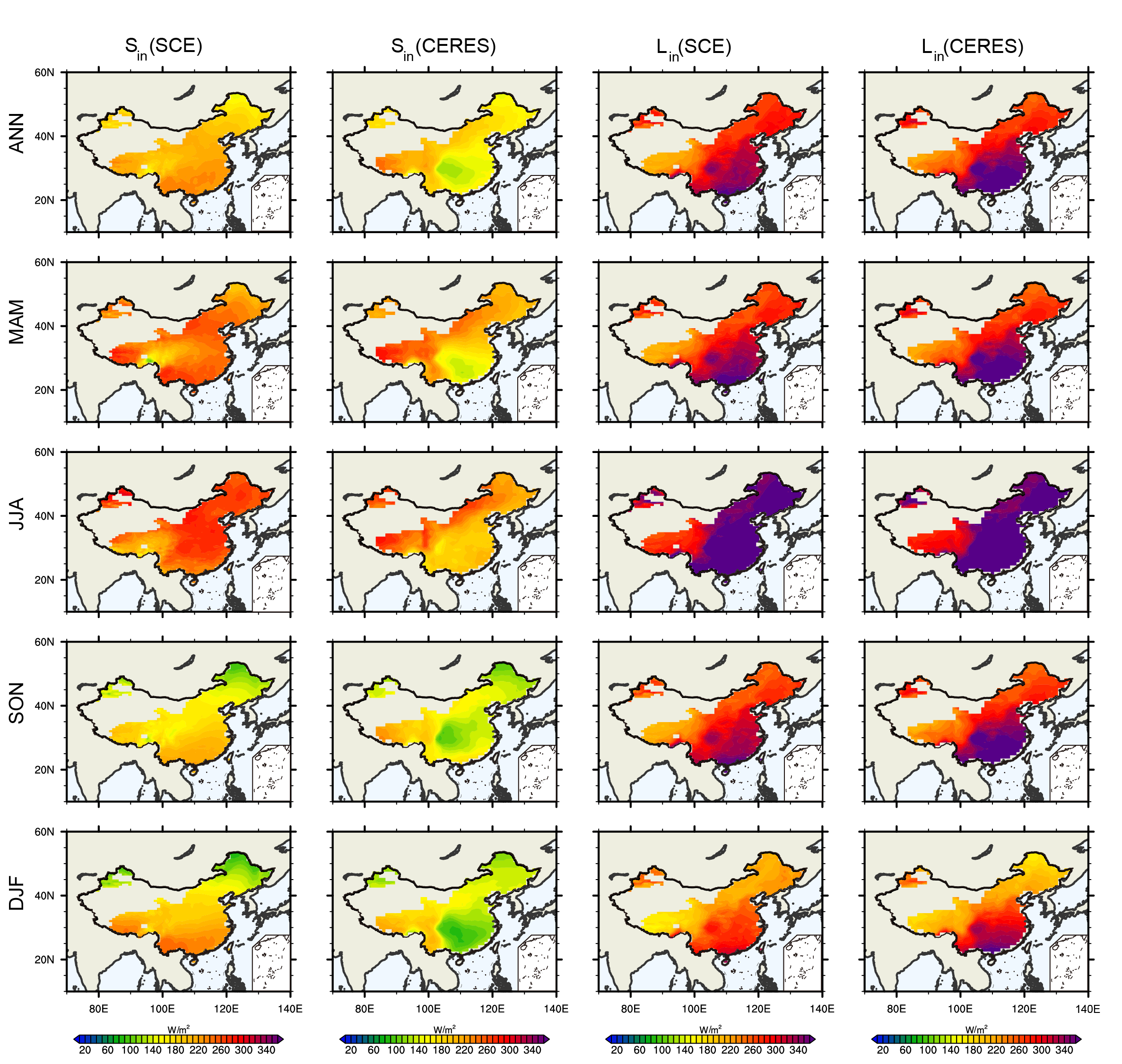
**

**Supplementary Fig. 3 Temporal changes of the anomalies in annual mean surface energy fluxes over China from 1982 to 2011. a**, downward shortwave radiation, **b**, downward longwave radiation, **c**, net shortwave radiation, **d**, net longwave radiation, **e**, latent heat flux, **f**, sensible heat flux. The black line represents the ensemble (30 members) mean from the experiment (“SCE”), and the red line denotes that from the control (“CTL”). The solid magenta (gray) and dashed green lines denote the observations or observation-based radiation from Clouds and the Earth’s Radiant Energy System (CERES) [34], observation-based ET products from Ref. [39] and Ref. [77]; output of energy flux from the European Centre for Medium-Range Weather Forecasts (ECMWF) reanalysis (ERA-Interim) [35] and National Centers for Environmental Prediction (NCEP) reanalysis data set [36].

**
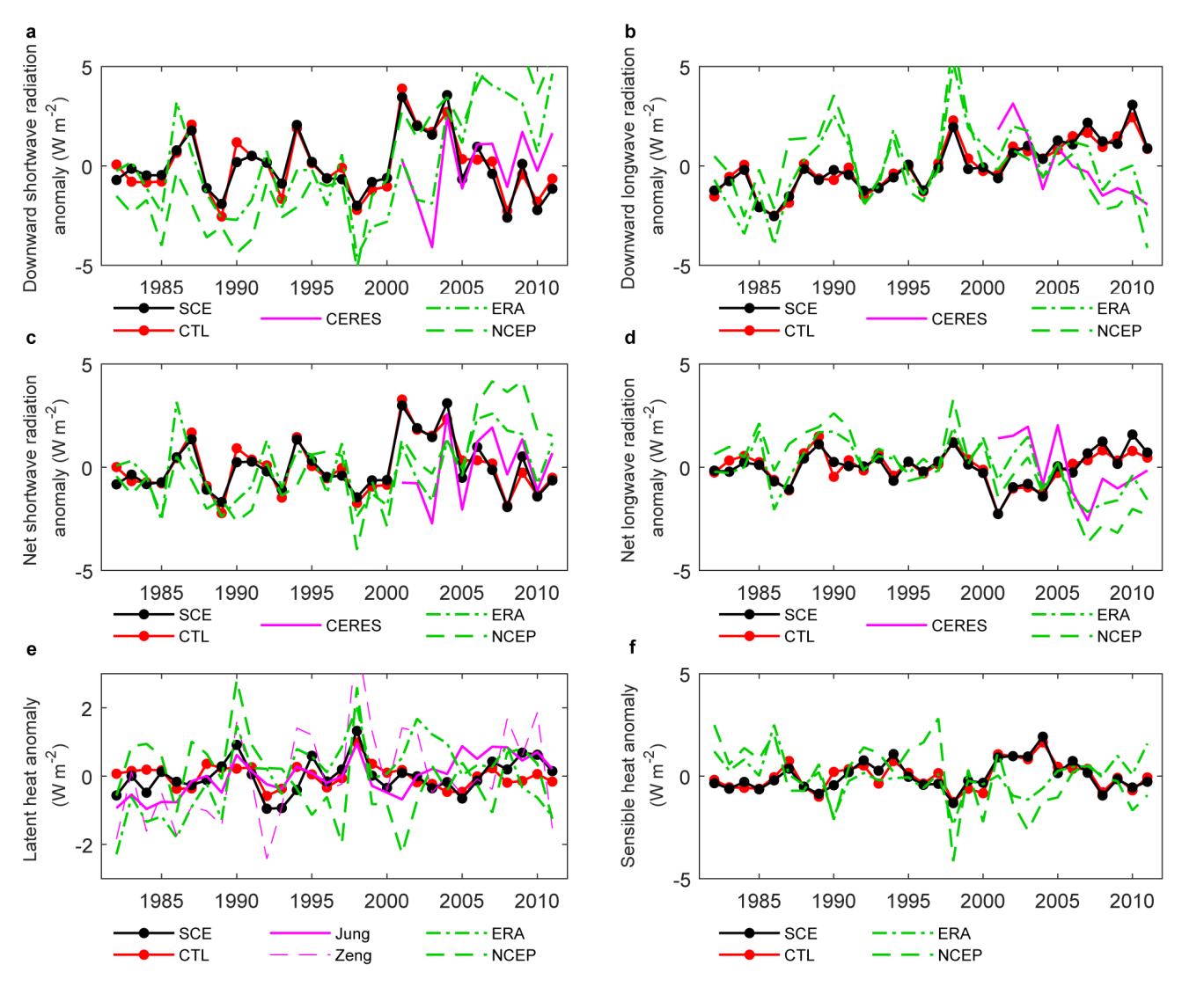
**

**Supplementary Fig. 4 Spatial patterns of the vegetation-induced trend in each component in the decomposition. a**, trend in reconstructed surface air temperature (Ta_rec); **b**, reciprocal of the energy redistribution (*f*) factor; **c**, trend in vegetation-induced combined forcing and response (CF), including surface climate forcing due to change in **d**, surface shortwave albedo (α); **e**, evapotranspiration (ET); **f**, surface aerodynamic resistance (r_a_); **g**, shortwave radiation (SW); **h**, air emissivity (ε_a_); **i**, sum of α and ET. All components are computed based on equation (8) in Decomposition of trend in surface air temperature (Methods). Trend of each component was computed for SCE minus CTL. The area with climatological leaf area index less than 0.1 was masked and shaded area indicates a 95% confidence level.


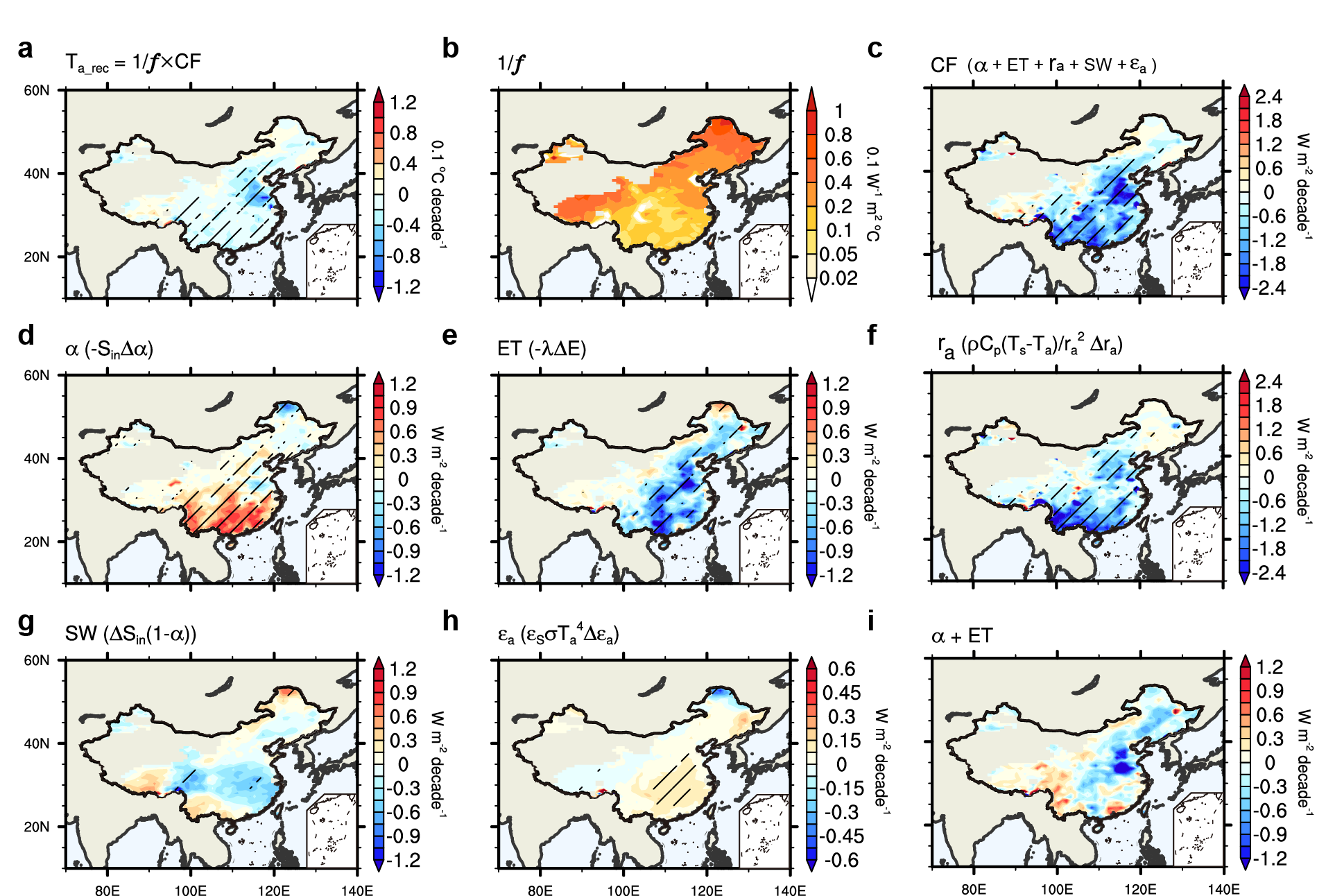


**Supplementary Fig. 5** Same as Figure 3g−l, but for spring (March, April and May, MAM) with the spatial pattern of vegetation-induced trend in surface relative humidity at 2m height (Q2m), column-integrated precipitable water (overlaid by the trend in wind at 850hPa) and total cloud cover additionally shown here.


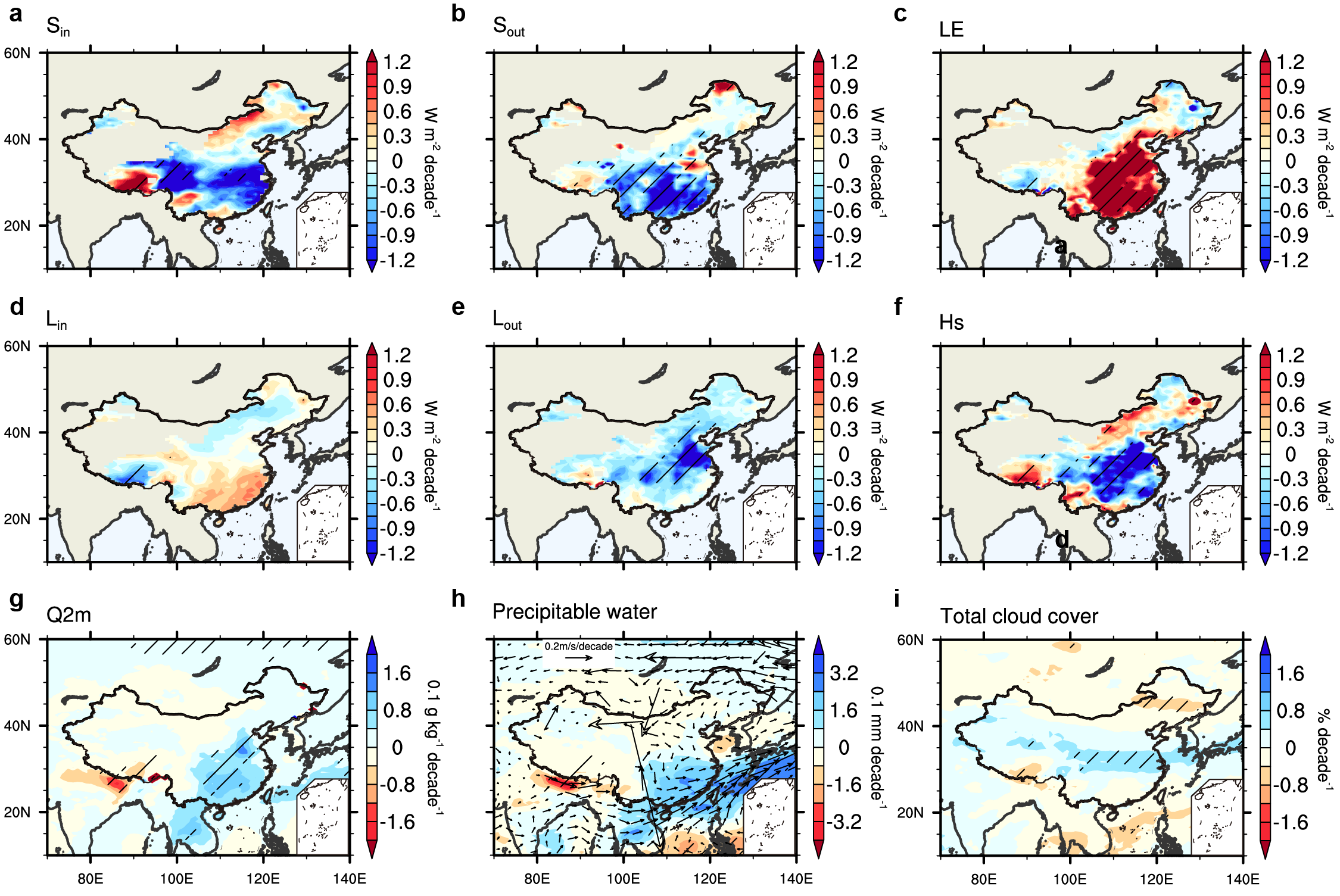


**Supplementary Fig. 6** Same as Figure 3g−l, but for summer (June, July and August, JJA) with the spatial pattern of vegetation-induced trend in surface relative humidity at 2m height (Q2m), column-integrated precipitable water (overlaid by the trend in wind at 850hPa) and total cloud cover additionally shown here.


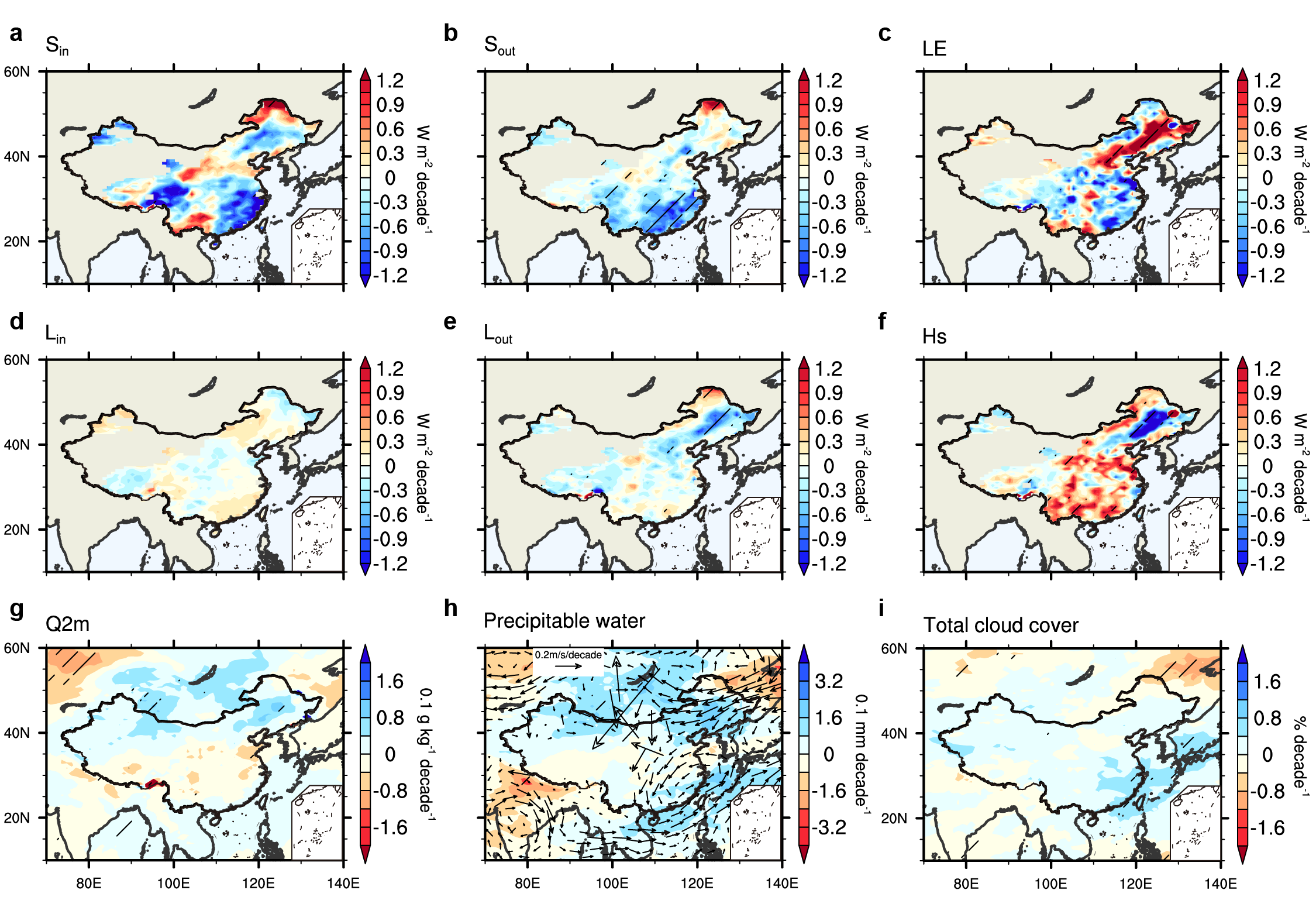


**Supplementary Fig. 7** Same as Figure 3g−l, but for autumn (September, October and November, SON) with the spatial pattern of vegetation-induced trend in surface relative humidity at 2m height (Q2m), column-integrated precipitable water (overlaid by the trend in wind at 850hPa) and total cloud cover additionally shown here.


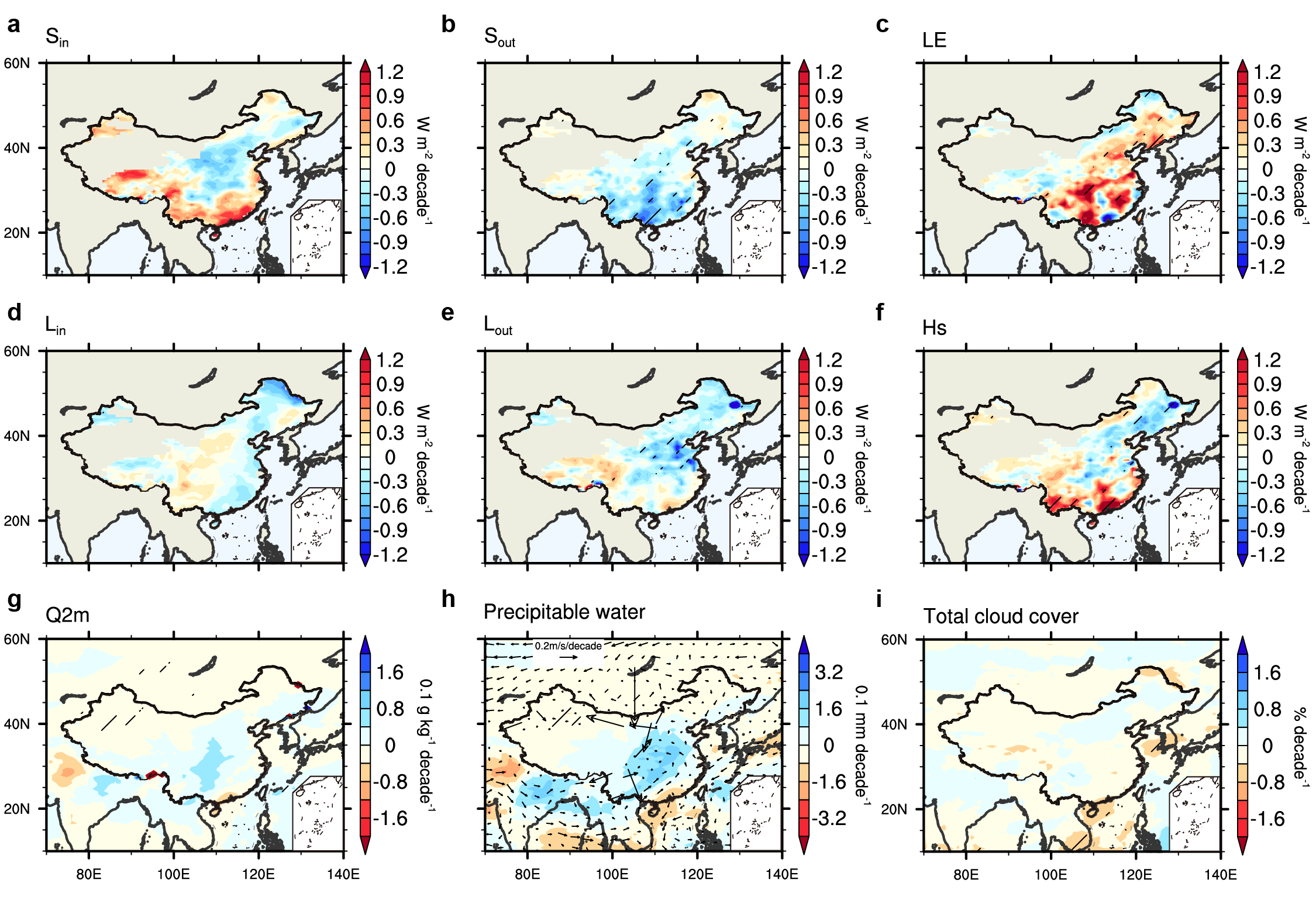


**Supplementary Fig. 8** Same as Figure 3g−l, but for winter (December, January and February, DJF) with the spatial pattern of vegetation-induced trend in surface relative humidity at 2m height (Q2m), column-integrated precipitable water (overlaid by the trend in wind at 850hPa) and total cloud cover additionally shown here.


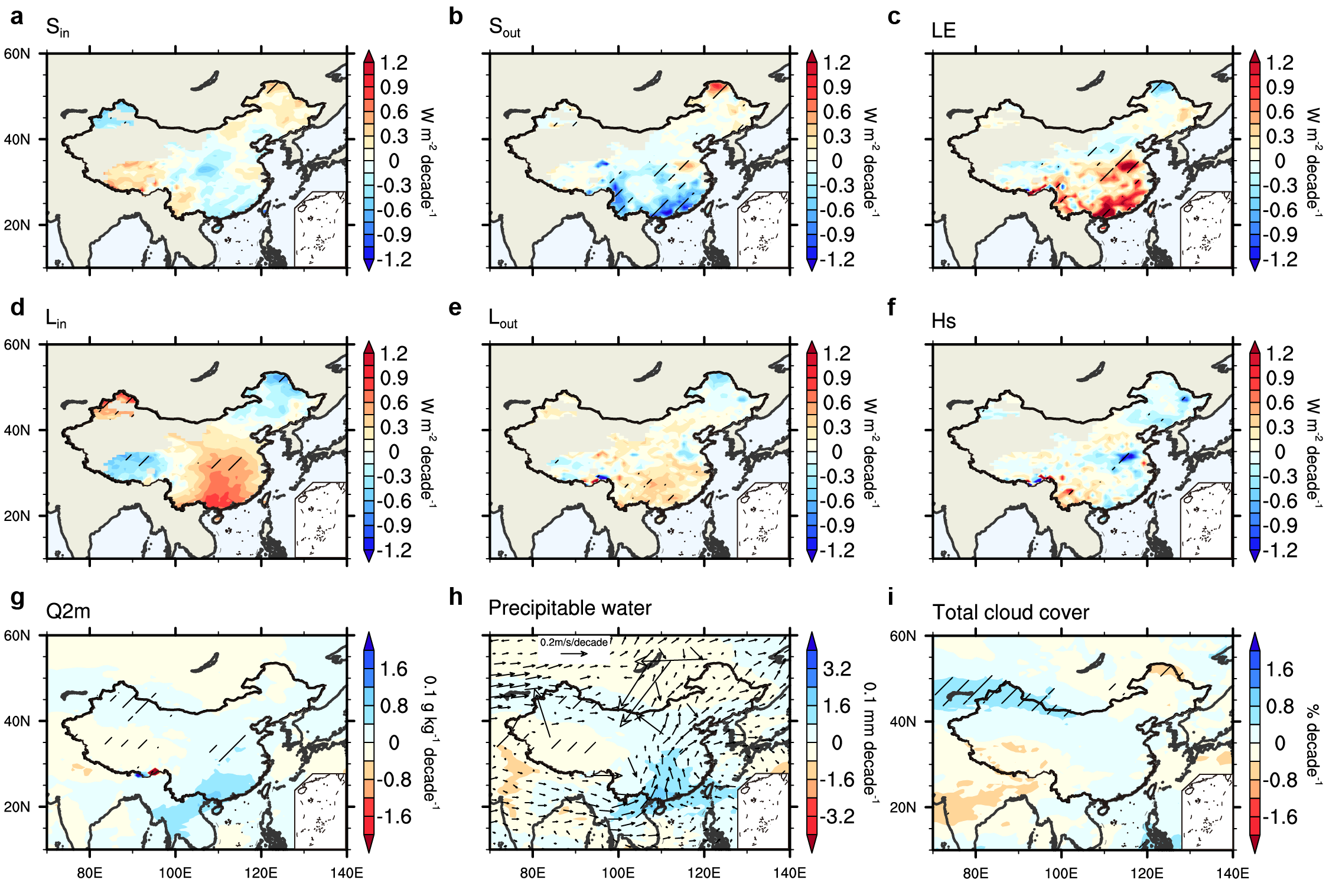


**Supplementary Fig. 9** Same as Figure 6, but for summer (June, July and August, JJA).

**
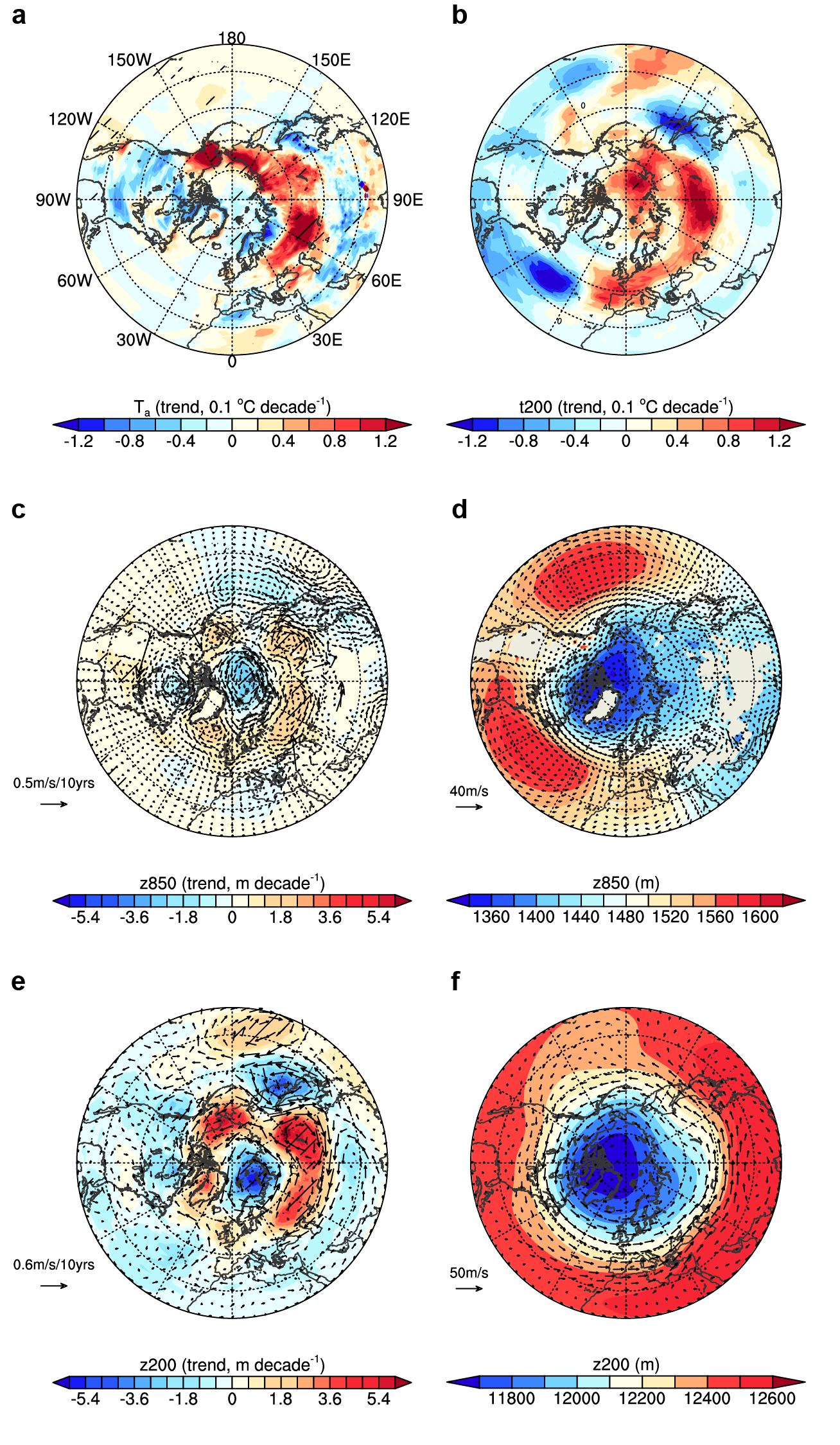
**

**Supplementary Fig. 10** Same as Figure 6, but for autumn (September, October and November, SON).

**
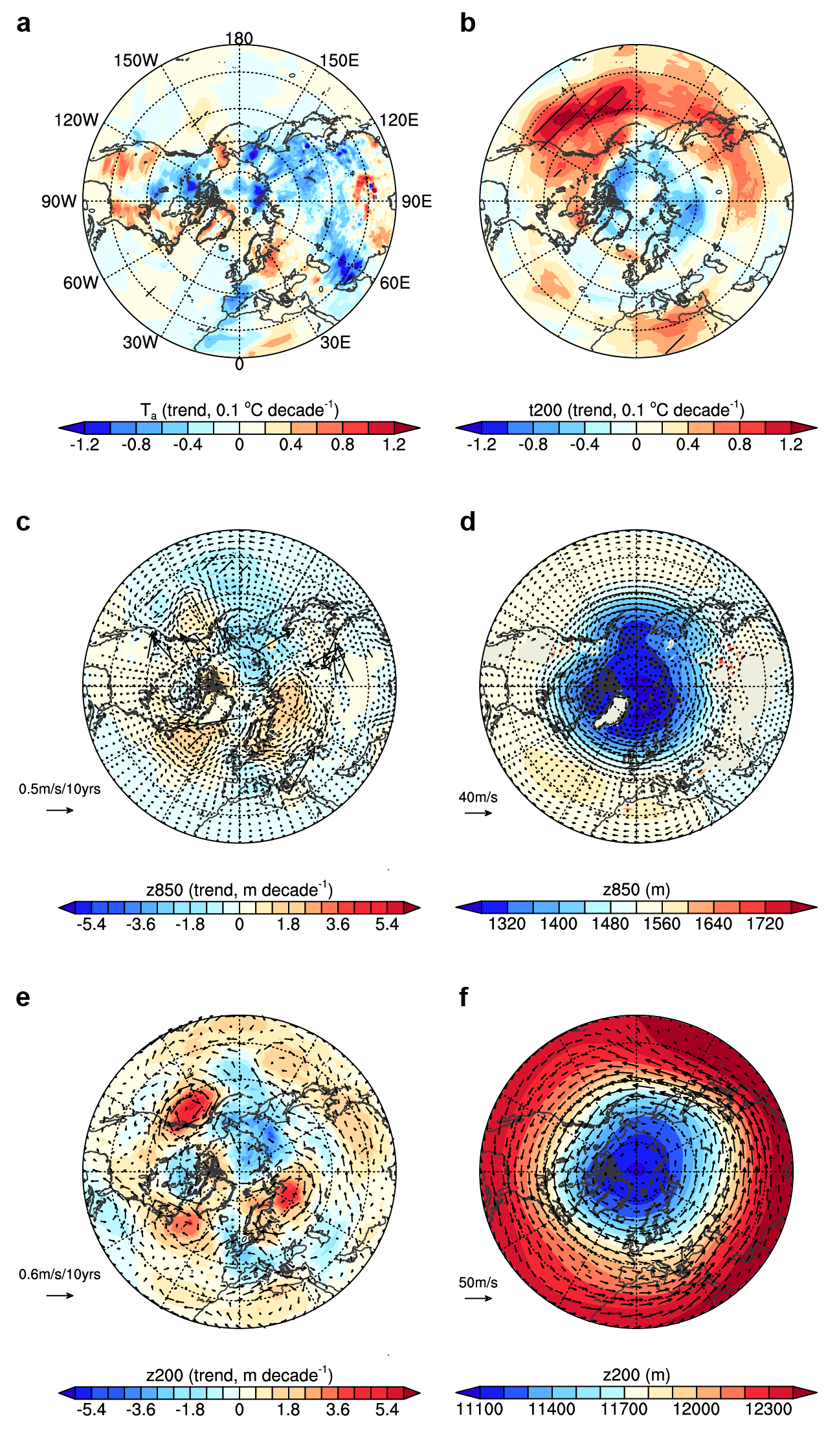
**

**Supplementary Fig. 11** Same as Figure 6, but for winter (December, January and February, DJF).

**
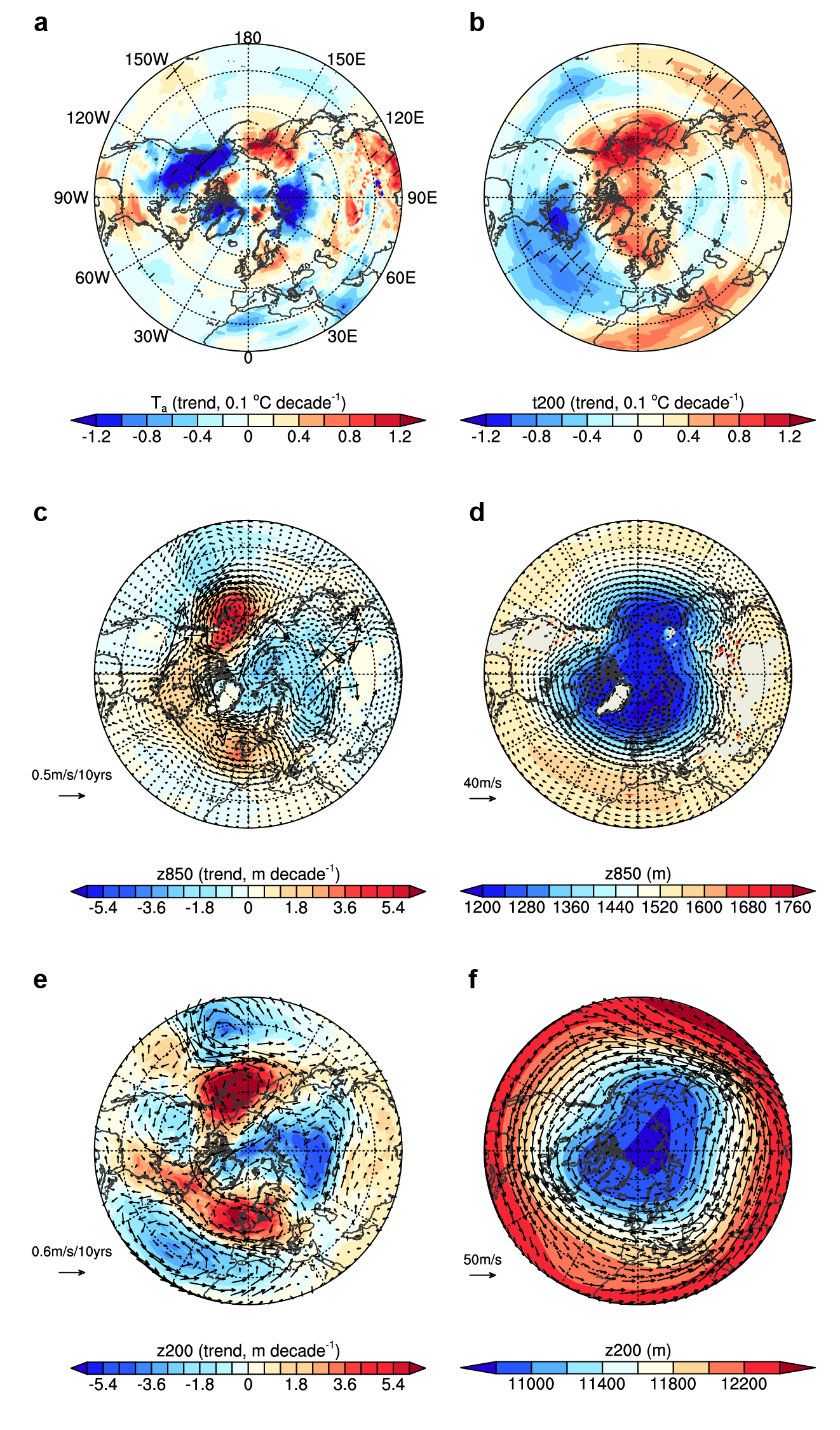
**

**Supplementary Fig. 12 Simulated trend patterns of global surface air temperature in boreal winter for 2002−2011.** The surface air temperature (T_a_) was derived from the SCE experiment and further averaged for boreal winter (December, January and February, DJF). The trend was calculated for 2002−2011 with the dotted area indicates a 95% confidence level.

**
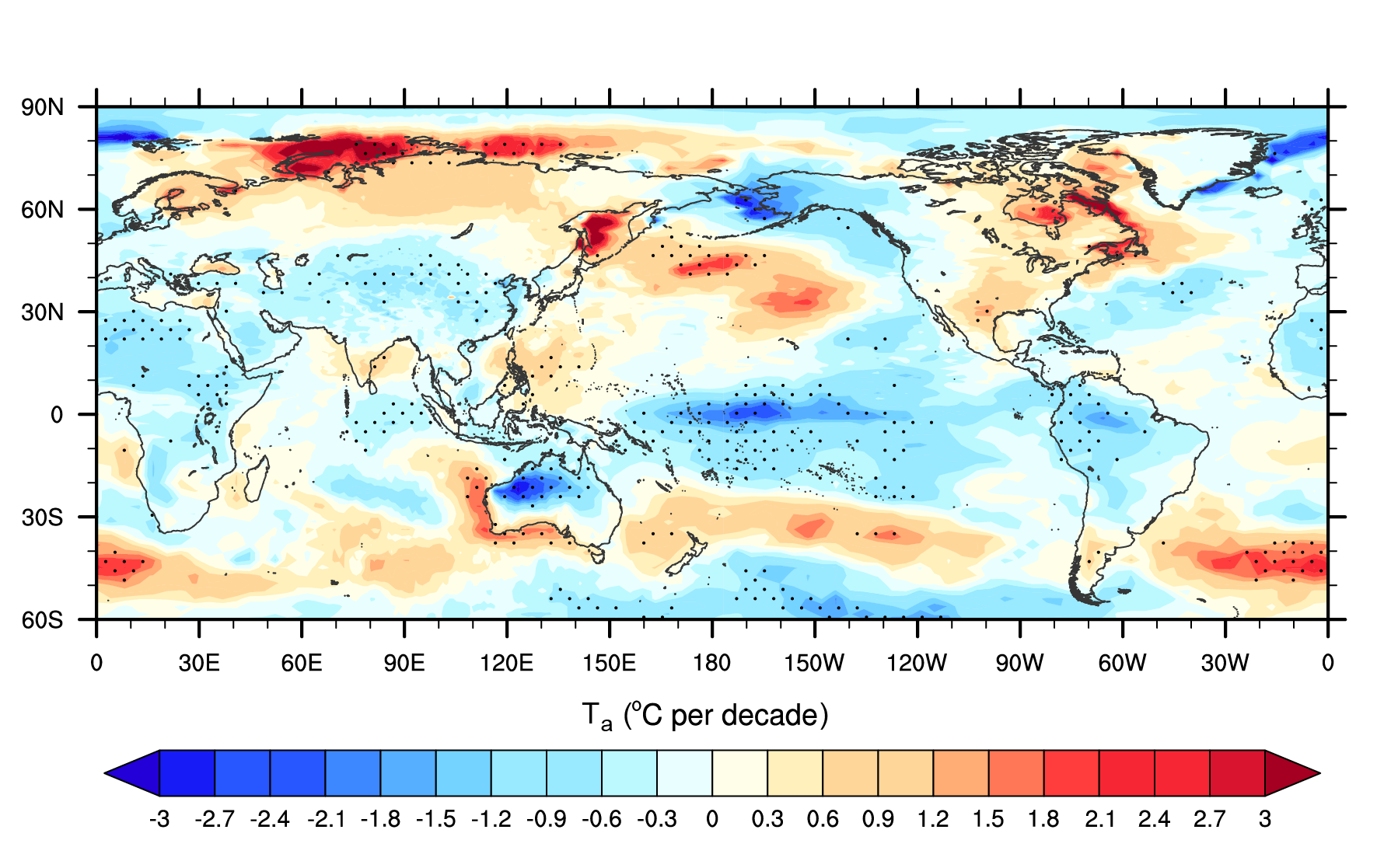
**

**Supplementary Fig. 13 Schematic diagram showing the difference in afforestation−induced change in surface temperature between local and large scale.** Land surface temperature 1 (LST1) was assumed to be the mean temperature on the surface of grassland, while LST2 was assumed to be surface temperature over the planted forest. n% indicates the percentage of planted forest over the domain. Since the difference between LST2 and LST1 indicates the biophysical effects of assumed 100% afforestation for the local-scale comparisons [8], this climate effects would be dampened over the domain at a large spatial scale.


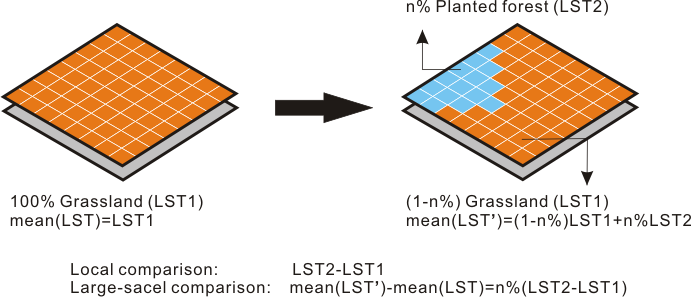


**Supplementary Fig. 14** **Change in land surface temperature and its decomposition shown by Lee’s and the modified method.** Detailed derivation processes could be seen in the Methods. ***Significance at the 99% confidence level, **Significance at the 95% confidence level, *Significance at the 90% confidence level. NS indicates not significant.


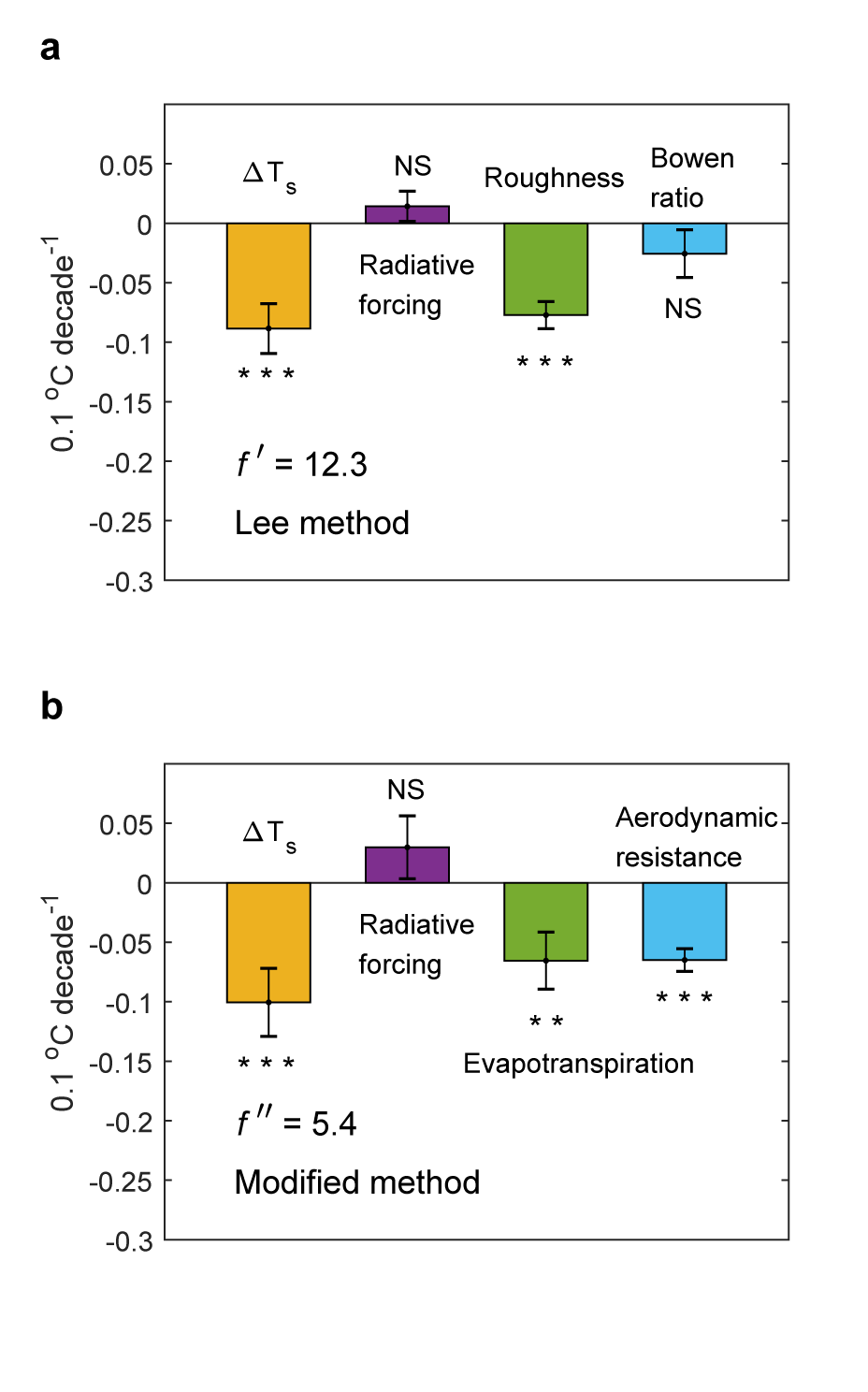


**Supplementary Fig. 15** **Vegetation-induced changes in controls on spring evapotranspiration (ET). a, c,** Correlation (ρ) between spring ET and precipitation (P); **b, d,** and between spring ET and surface net radiation (R_n_) during 1982−2011. Results from CTL experiment are shown in the left column and SCE experiment in the right column. Shaded area with black dots indicates a 95% confidence level. For both CTL and SCE simulations, significant positive correlation between ET and P indicate the water-limited regimes over the semi-arid and arid regions while the positive correlation between ET and R_n_ indicates an energy-limited regime in southern China.


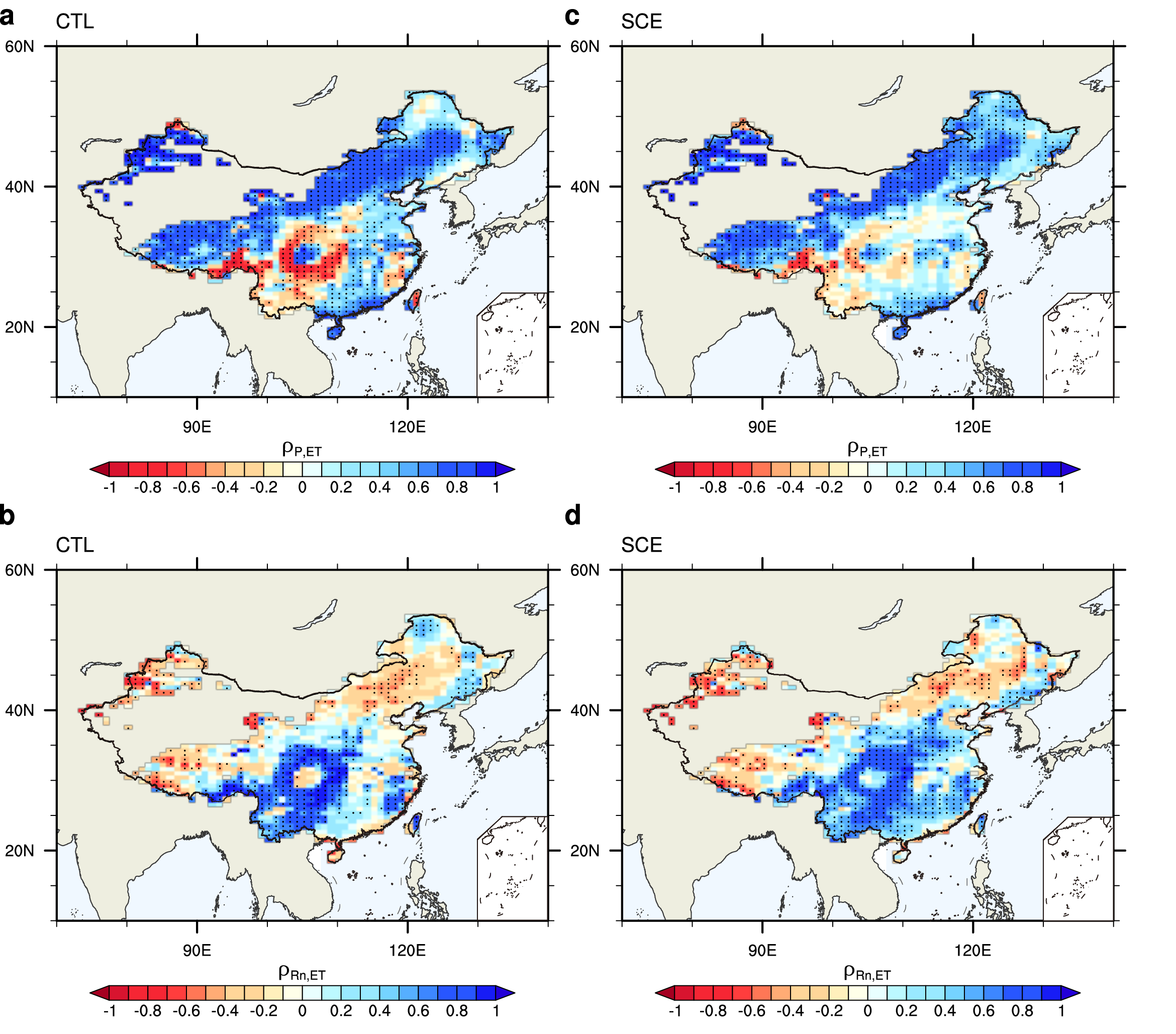


**Supplementary Fig. 16** **Spatial patterns of vegetation-induced trend in extreme temperature indices. a**, **b**, **c**, **d**, **e**, **f**, spring (March, April and May, MAM); **g**, **h**, **i**, **j**, **k**, **l**,, summer (June, July and August, JJA); **m**, **n**, **o**, **p**, **q**, **r**, fall (September, October and November, SON) and **s**, **t**, **u**, **v**, **w**, **x**, winter (December, January and February, DFJ). Indices for monitoring extreme temperature include warm days (TX90p), cool nights (TN10p), summer days (SU25), warm spell duration indicator (WSDI), frost days (FD0) and cold spell duration indicator (CSDI), which were selected from the list that is recommended by Expert Team on Climate Change Detection and Indices (ETCCDI). Definitions of these indices are described in detail in Ref. [67]. Note that for TX90p, TN10p, WSDI and CSDI, the 10th and 90th percentiles of daily maximum/minimum temperature are determined from CTL experiments during the 1982−2011 base period. Based on the CTL-calculated percentiles, extreme temperature indices in each season were first computed for both SCE and CTL experiments and for each year from 1982 to 2011. The linear trend of SCE minus CTL was further computed and shown in the figures above. The area with climatological leaf area index less than 0.1 was masked and hatching indicates a 95% confidence level.


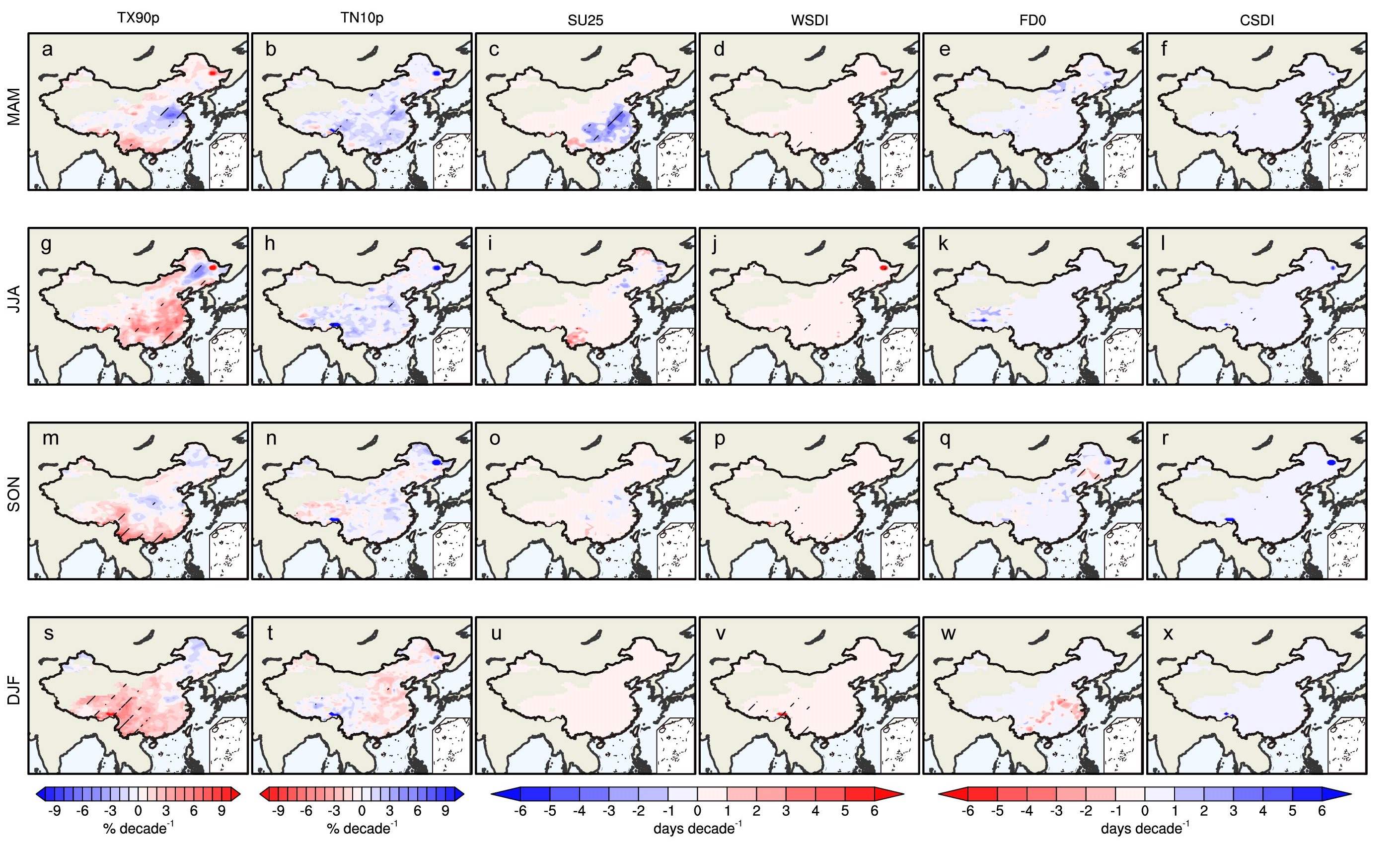

Supplement: nwz132_Supplemental_File [file nwz132_supplemental_file.doc]
